# Supplementary material for: quantms: a cloud-based pipeline for quantitative proteomics enables the reanalysis of public proteomics data
Source: Nat Methods. 2024 Jul 4;21(9):1603–7. doi: 10.1038/s41592-024-02343-1 (PMC11399091; doi:10.1038/s41592-024-02343-1)
Supplement: Supplementary file 1 — Supplementary Notes 1–10, Figs. 1–24 and Tables 1–10. [file 41592_2024_2343_MOESM1_ESM.pdf]

nature methods

Supplementary information https://doi.org/10.1038/s41592-024-02343-1

quantms: a cloud-based pipeline for quantitative proteomics enables the reanalysis of public proteomics data

In the format provided by the authors and unedited

Supplementary Note 1: Peptide/protein identification using multiple search engines. *2*

Supplementary Note 2: Label-free benchmark datasets. *4*

PXD001819: Spike-in UPS Label-Free dataset. 4

Supplementary Note 3: TMT benchmark datasets. *8*

PXD005486: Statistical Models for the Analysis of Isobaric Tags Multiplexed Quantitative Proteomics. 8

PXD002875: Proteome-wide quantitative multiplexed profiling of protein expression: Carbon source dependency in S. cerevisiae. 11

PXD007683: TMT vs LFQ benchmark using quantms. 11

Supplementary Note 4: Large-scale datasets analysis *15*

Supplementary Note 5: DIA benchmark datasets. *16*

PXD026600: DIA analysis of the UPS1 E. coli proteomic standard 16

Supplementary Note 6: Single cell benchmark datasets. *20*

Supplementary Note 7: Data reanalysed with quantms. *27*

Supplementary Note 8: IBAQ-based expression profile provided by quantms.org *41*

Supplementary Note 9: Quality control using pmultiqc. *42*

Peptides Quantification Table and Protein Quantification Table: Searching specific peptides and proteins 43

Supplementary Note 10: Implementing workflows on AWS, Google Cloud and other cloud infrastructures. *45*

References *46*

Supplementary Note 1: Peptide/protein identification using multiple search engines.

quantms enables the identification of peptides and proteins in data-dependant acquisition experiments using multiple search engines. By February 2023, the workflows supported two major search engines MSGF+ (version v2021.03.22) [1] and comet (version 2019.01 rev. 5) [2]. Documentation on how to configure the search engines can be found at https://quantms.readthedocs.io/en/latest/identification.html. As shown in previous studies, the use of multiple search engines can increase the number of peptide spectrum matches, peptides and proteins identified [3, 4]. Figure 1 shows the number of PSMs for the PXD004683 reanalysis as reported by pmultiqc in the Spectra Tracking section (the full report can be seen here: http://ftp.pride.ebi.ac.uk/pub/databases/pride/resources/proteomes/differential-expression/PXD004683/summarypipeline/multiqc_report.html). For this experiment MSGF+ identified for all MS runs almost 5% more PSMs than Comet. Multiple search engines are extensively used in studies where deep coverage of the proteins is desired (e.g., proteogenomics experiments [5]). In addition, we observed another major advantage of supporting multiple search engines during reanalysis: two search engines can compensate for one search engine performing suboptimal or even failing on some datasets or individual MS runs.

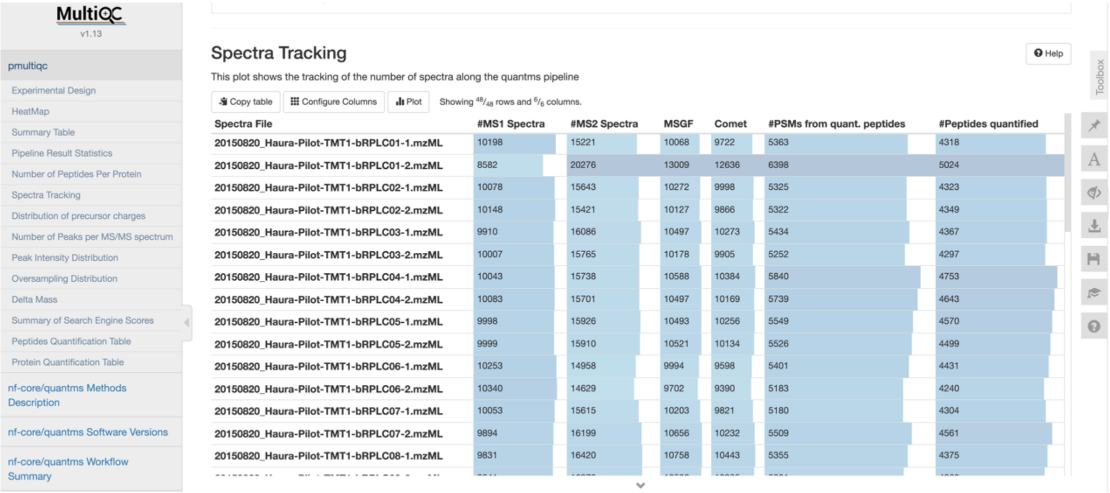

Figure 1: Number of peptides identified by Comet and MSGF+ for reanalysis of dataset PXD004683. The report of the number of PMSs by the search engines is part of the pmultiqc, section Spectra Tracking.

quantms parallelize the identification step for each MS run and each search engine combination allowing to scale of the identification process over the size of the experiment and the number of search engines used. However, using several search engines requires more computational resources and the overall processing time for identification is dominated by the slowest-performing search engine when analyzing an experiment. Figure 2 shows the memory usage at each step (tool) of quantms during the analysis of PXD004683 (the full report can be found http://ftp.pride.ebi.ac.uk/pub/databases/pride/resources/proteomes/differential-expression/PXD004683/pipeline_info/execution_report_2022-11-22_18-15-55.html). In this experiment, MSGF+ consumes two times more memory than Comet search engine (on average the MSGF+ nodes needed 12 GB memory, while Comet in average needed 4GB). MSGF+ is 5 times slower (see execution report) than Comet due to its more complex scoring approach and dominates the computational time and the resources allocated for the whole quantification workflow. We are currently evaluating the inclusion of additional search engines that yield similar performance at lower memory requirements or faster search.

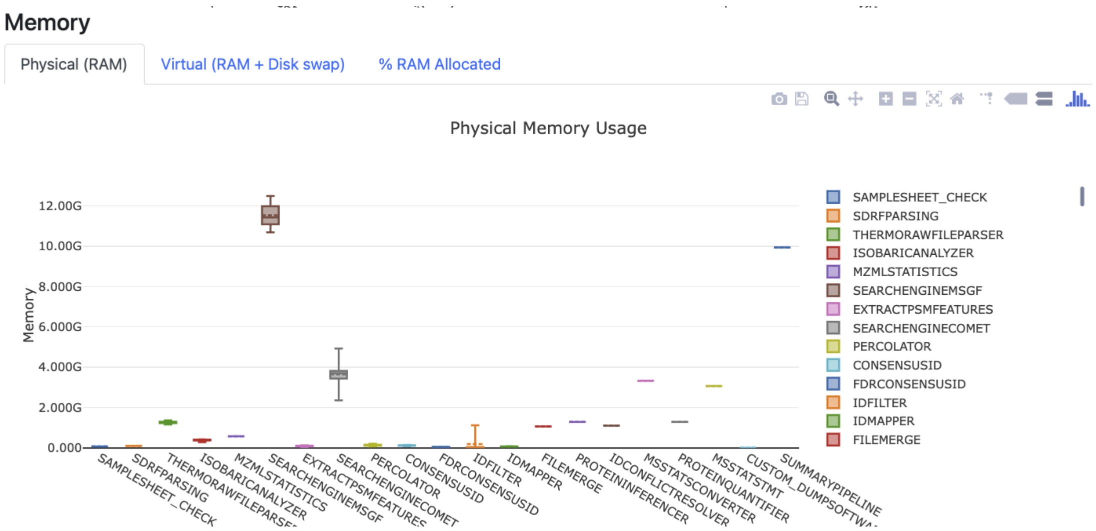

Figure 2: Physical memory usage by all the steps of the analysis of project PXD004683 as reported by the nf-core execution report.

Supplementary Note 2: Label-free benchmark datasets.

The MaxQuant software is used by major groups performing public data reanalysis (e.g., MassIVE.quant [6], ProteomicsDB [7] and PRIDE Archive with ExpressionAtlas [8, 9]). To ensure that no major flaws in our implementation exist that critically affect quantification performance we benchmarked MaxQuant (version 1.6.10.43) and quantms label-free workflow on multiple datasets and compared quantification performance and results. Search parameters, including modifications, were set according to the original description in each manuscript. The Uniprot-Swissprot reviewed database without isoforms (version 10.20222) was used. The choice of tools for differential expression analysis is based on a previous comparison of different R-packages in combination with MaxQuant and quantms [10].

PXD001819: Spike-in UPS Label-Free dataset.

The spike-in UPS dataset **PXD001819** [11, 12] has been used in multiple benchmark studies [11, 13, 14]. It contains 48 Sigma UPS1 proteins spiked into a background of yeast cell lysate in nine different concentrations: 0.05, 0.125, 0.25, 0.5, 2.5, 5, 12.5, 25 and 50 fmol/ul. The parameters for the quantms analysis were derived from the initial publication [11]. We compare the quantms results with the peptide and protein quantified by MaxQuant in the original publication [12]. The Jupyter notebook with the benchmarking results and code to reproduce the figures can be found at https://github.com/ypriverol/quantms-research/blob/main/notebooks/LFQ-DDA/PXD001819Benchmark.ipynb

Table 1: Three UPS datasets reanalysis using quantms LFQ pipeline. MaxQuant results were obtained from the original manuscript [13].

Tools	Proteins quantified	Proteins quantified Spike-in proteins detected, n (%)	Proportion of missing values in the detected spike-in proteins (%)	Proportion of missing values in the detected background proteins (%)
**quantms**	1144	48 (100%)	2.2	4.6
**MaxQuant**	1063	48 (100%)	31.0	5.3

Table 1 shows the comparison between quantms and MaxQuant as reported by *Palomba et. al* [13]. quantms identified and quantified more proteins than MaxQuant including all 48 UPS spike-in proteins and the number of missing values in the background proteins was higher in quantms than MaxQuant. A comparison between the results of the quantms pipeline and the median MaxQuant intensities across replicates (**Figure 3**) reveals that up to a spike-in concentration of 2500 amol, both methods perform similarly in fold change accuracy, except that quantms quantified UPS proteins with less missing values. The proteins with at least 50% measurements in the replicates group are considered. In the lower concentration range, MaxQuant loses quantifiable proteins quicker than quantms but if it reports a fold change it determined the true fold change more accurately. In contrast, quantms consistently quantify more proteins but can underestimate the true fold changes, especially when the lower spike-in concentration in the comparison was 500 amol or lower.

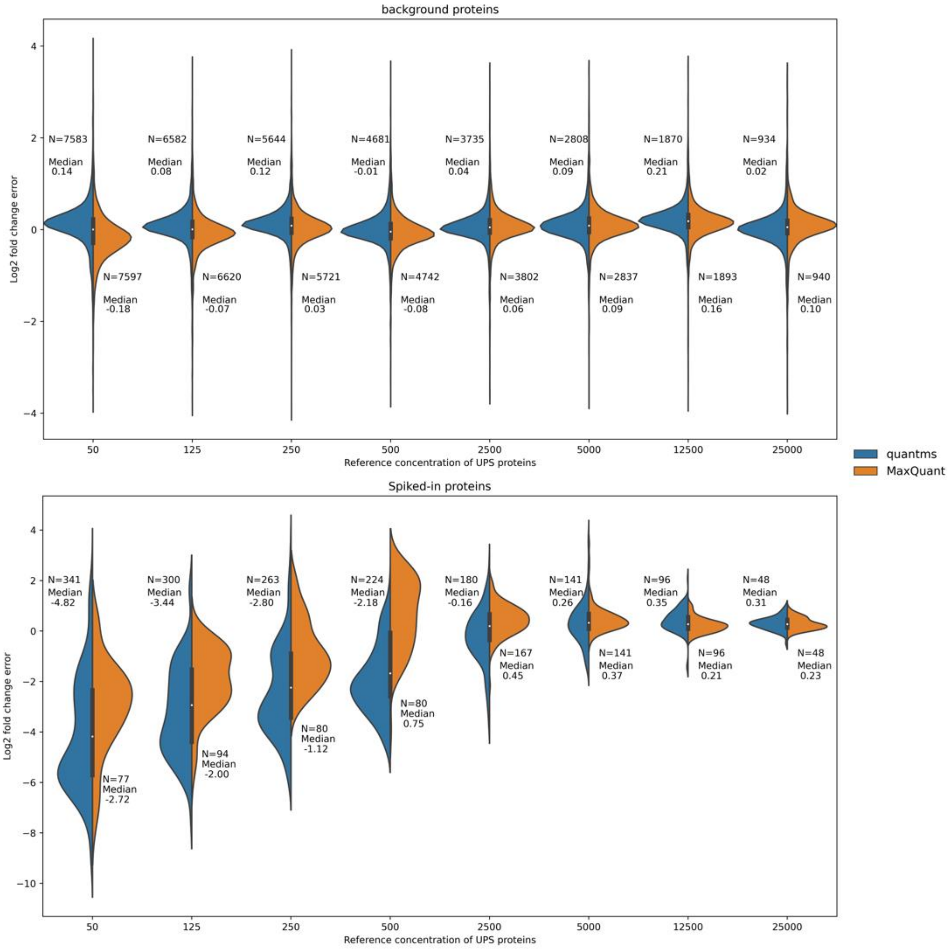

Figure 3: Values are summarized over all possible comparisons for a reference concentration for better readability. At a given reference concentration, all possible comparisons are included. **Top:** Violin plots of log2 fold change errors (closer to 0 = better) from background yeast proteins. The error is calculated as observed fold change minus expected, therefore errors greater than zero mean overestimation. **Bottom:** Violin plots of log2 fold change errors

from UPS proteins. The number of comparisons and error median is provided. Source data are provided in the supplementary data file. Box, median ± interquartile range; whiskers, 1.5× interquartile range; bounds of box, minima, maxima.

While most of the benchmarks in this manuscript will be performed between quantms and MaxQuant, we also add a Venn diagram comparing with other tools benchmarked by *Palomba et. al.* [11]. The original manuscript uses two other tools, Skyline [15] and Mascot-MFPaQ [16] in combination with MaxQuant with two configurations LFQ and Raw Intensity (summed peptide intensity values) (**Figure 4**). All workflows share more than 70% of quantified proteins, quantms and MFPaQ are the tools that quantified more unique proteins, 241 and 180 proteins respectively.

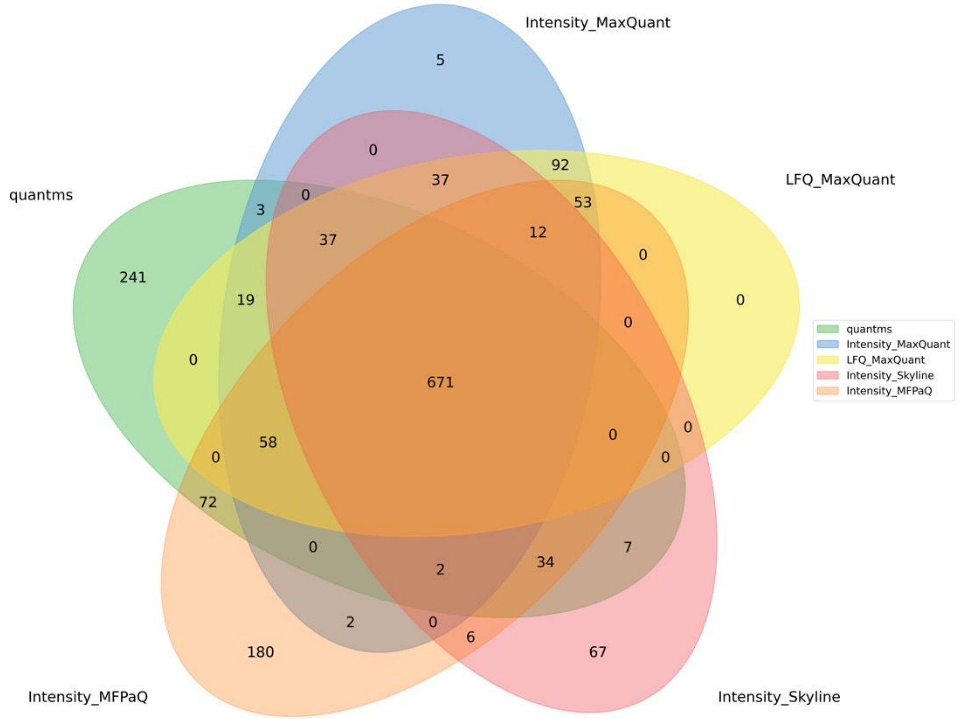

Figure 4: Venn diagram of quantified proteins by three different tools quantms, MaxQuant (two combinations, Intensity and LFQ), Skyline, and Mascot-MFPaQ (MFPaQ).

To measure the variability of protein quantities between replicates, we calculate the coefficients of variation (CV), considering only proteins that are quantified in at least 50% of the replicates. Our analysis revealed that the median CVs obtained using quantms are smaller (less than 4%) compared to those obtained with MaxQuant when the amount of protein quantified is consistent (**Figure 5**).

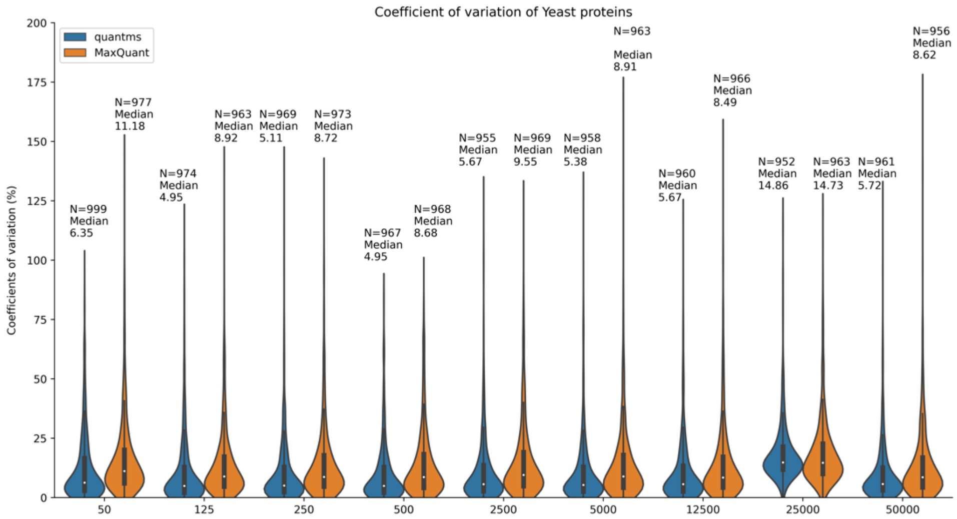

Figure 5: Coefficient of variation of Yeast proteins for the reanalysis of PXD001819 using MaxQuant and quantms. Source data are provided in the supplementary data file. Box, median ± interquartile range; whiskers, 1.5× interquartile range; bounds of box, minima, maxima**.** The number of yeast proteins and median coefficient of variation are provided.

Supplementary Note 3: TMT benchmark datasets.

We used three datasets PXD005486 [17], PXD002875 [18], and PXD000768 [19] to benchmark quantms with MaxQuant or the original results from the studies.

PXD005486: Statistical Models for the Analysis of Isobaric Tags Multiplexed Quantitative Proteomics.

In 2017, D' Angelo *et. al.* [17] published a dataset of E. Coli background with 12 spike-in human proteins and a bovin protein in multiple known concentrations. The analytical method employed tandem mass tags (TMT), and proteins were spiked twice using the same concentration in different channels and only once for the two highest concentrations. Therefore, only a single, or two replicate measurements at maximum are available when comparing two concentrations. A total of 12 peptide fractions were prepared. The complete description of the dataset can be found in the original manuscript [17]. The benchmark analysis can be found here: https://github.com/ypriverol/quantms-research/blob/main/notebooks/TMT/PXD005486Benchmark.ipynb

Like in the original study, we assigned the first five channels to treatment group one and the second five channels to treatment group two. For MSstatsTMT [20], global normalization and MSstats summarization method are enabled. **Figure 6** shows the correlation of the reporter intensities for background proteins across all channels, and it indicates excellent sample-to-sample reproducibility (R^2 > 0.98 for all channel combinations).

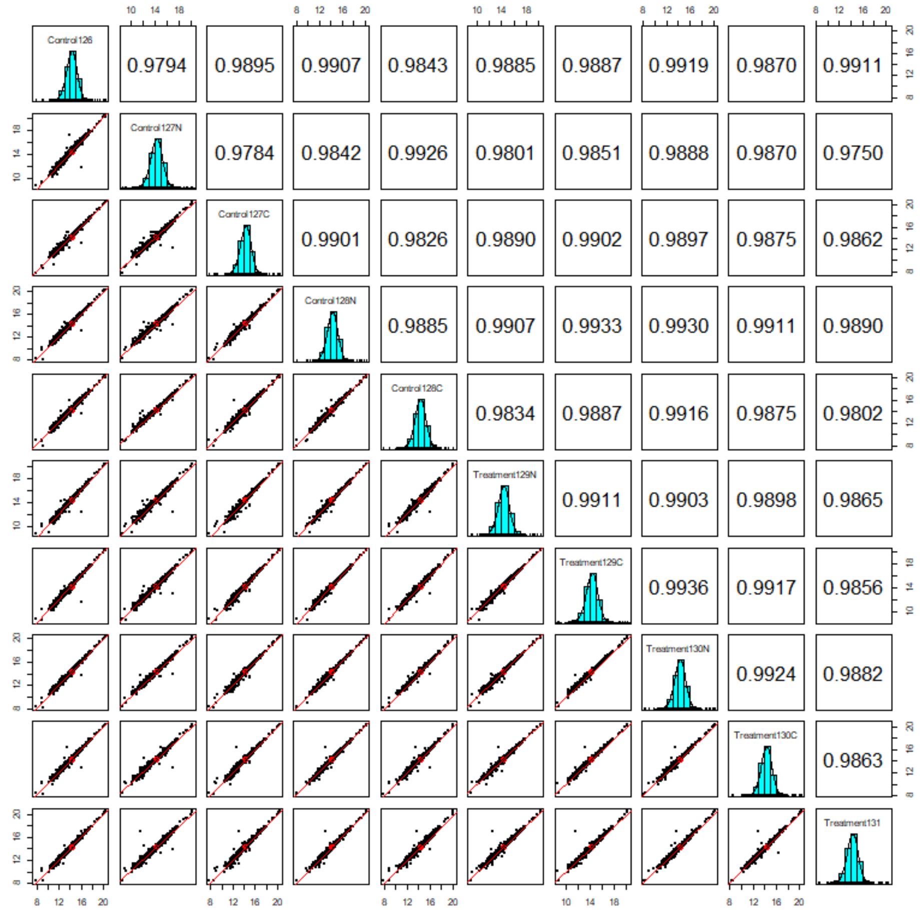

Figure 6: Correlation of reporter intensities of background proteins for all channels in PXD005486 TMT data set. The protein intensities are generated by MSstatsTMT. Global normalization and MSstats summarization methods are enabled within MSstatsTMT. Each subplot represents a different channel and Pearson correlations are shown.

This dataset allows us to assess how accurate fold-changes for spike-in proteins are recovered. quantms output generated with MSstatsTMT confirms that the distribution of observed fold-changes between unchanged background E. coli proteins is centred around the expected fold-change of zero (median=0.002, SD=0.07, see Figure 7).

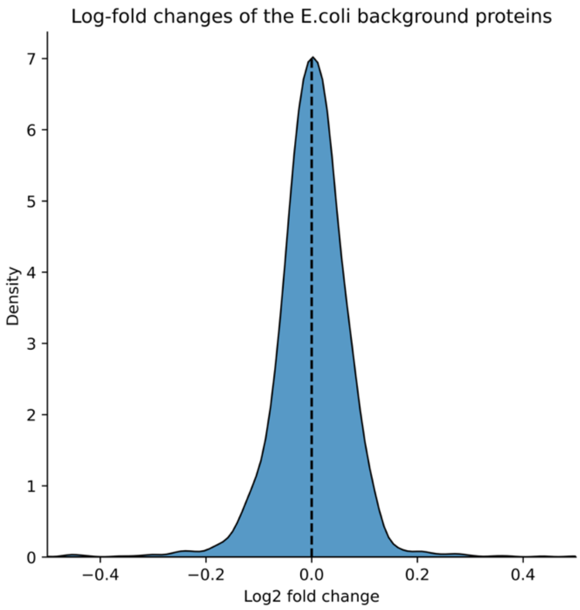

Figure 7: Log-fold changes of the E. coli background proteins obtained from the comparison of the first five channels against the other five ones (Estimated log-fold changes are centred around 0.

Table 3 shows the expected concentration, the observed bias, and root-mean-square error (RMSE) for all spike-in proteins for quantms workflow, IsoProt pipeline [21] and the best method from D' Angelo's original analysis [17]. The bias of the coefficient estimate is calculated by subtracting the true value from the estimated value for pairwise comparisons. Bias is often referred to as accuracy and can be considered a measure of ratio recovery. In all cases, quantms FCs have a lower RMSE for all concentrations. In addition, quantms achieves a slightly lower observed bias than the other workflows.

Table 3: Observed bias and RMSE of estimated fold-changes of the D'Angelo *et. al.* [17] and Johannes Griss *et. al.* [21] for dataset PXD005486.

Expected FC	**2**	**4**	**20**	**40**
Bias (quantms)	-0.5	-1.8	-9.7	-19.3
Bias (IsoProt)	-0.6	-2	-11.4	-22.7
Bias (ref)	-0.6	-1.8	-11.7	-21.9
RMSE (quantms)	0.555	1.85	10.1	19.9
RMSE (IsoProt)	1	2.1	11.8	23.5
RMSE (ref)	1	1.9	11.2	22.3

PXD002875: Proteome-wide quantitative multiplexed profiling of protein expression: Carbon source dependency in S. cerevisiae.

In 2015, Paulo JA *et. al.* [18] published a non-spiked TMT dataset to detect the global proteomic alterations in the budding yeast Saccharomyces cerevisiae due to differences in carbon sources in triplicate. All the data related to the benchmark can be found in a Jupyter notebook https://github.com/ypriverol/quantms-research/blob/main/notebooks/TMT/PXD002875Benchmark.ipynb.

Table 4 shows that the quantms workflow identified and quantified more peptides and proteins than MaxQuant. Overall, quantms achieves a lower median coefficient of variation (4.6%).

Table 4: Proteins quantified, and peptides identified by MaxQuant, quantms in the PXD002875 dataset.

	**Total quantified proteins**	**Total identified unique peptides**	**CV in galactose condition**	**CV in glucose condition**	**CV in raffinose condition**
quantms	4930	65323	5.2%	4.6%	5.6%
MaxQuant	4725	51606	6.6%	4.0%	7.1%
Shared	4481	/	/		

PXD007683: TMT vs LFQ benchmark using quantms.

We systematically compared two of the most common data-dependent workflows for proteome-wide quantitation, isobaric labelling with tandem mass tags (TMT) and label-free (LFQ) with Match-Between-Runs between MaxQuant and quantms using the ProteomeXchange dataset PXD007683. All the data related to the benchmark can be found in a Jupyter notebook https://github.com/ypriverol/quantms-research/blob/main/notebooks/TMT/PXD007683Benchmark.ipynb.

Yeast lysate is spiked into human lysate to 10% of total protein concentration (1× group), 5% (2× group), and 3.3% (3× group) for a total of 11 samples in PXD007683 so that the ratio between the yeast proteins in the first group and second has an expected fold change of two. In **Table 5,** we summarize the two data sets, running with MaxQuant and quantms. The TMT workflow quantified 10% more total proteins and 15% more yeast proteins by quantms, and the LFQ workflow quantified 11%

more total proteins and 23% more yeast proteins. On the other hand, the TMT method increased the total number of quantified proteins and peptides compared to the LFQ method.

Table 5: Proteins and unique peptides quantified by MaxQuant, quantms in PXD007683 dataset, including LFQ and TMT.

	**Total quantified proteins in TMT**	**Yeast proteins in TMT**	**Total quantified unique peptides in TMT**	**Total quantified proteins in LFQ**	**Yeast proteins in LFQ**	**Total quantified unique peptides in LFQ**	**Shared protein**
**quantms**	9415	1462	77341	8317	1151	53214	7653
**MaxQuant**	8486	1238	57838	7455	938	54473	6695
**Shared**	8112	1151	55679	7099	852	47237	/

Figure 8 shows quantification results in quantms MaxQuant and quantms with MSstats/MSstatsTMT in label-free (left panel) and TMT (right panel) experiments, respectively. Only proteins quantified with at least 50% measurements are considered. When MSstats is used including equalizeMedians normalization, Tukey's median polish estimation method and imputation parameters, the accuracy of estimation of log2FC is significantly improved than quantms with summed intensity. The quantms with MSstats achieved lower root mean square error on the estimation of fold change than MaxQuant with raw protein intensities. For MSstatsTMT, the global normalization and MSstats summarization methods to protein level are selected. Almost all tools achieved relatively high levels of accuracy (within 10% of expected for fold changes).

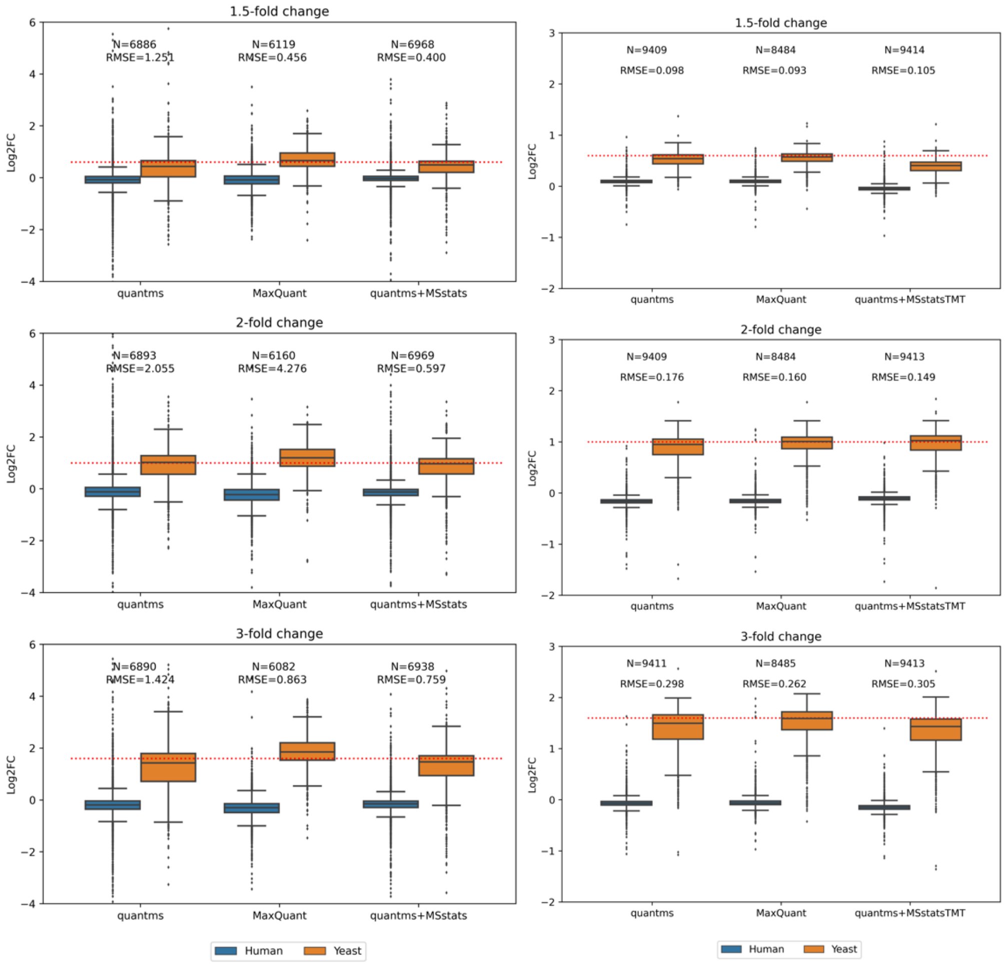

Figure 8: Boxplots of the distribution of fold change are shown for 1.5-fold change, 2-fold change and 3-fold change on label-free (left panel) and TMT (right panel) experiments from PXD007683, relatively. Box, median ± interquartile range; whiskers, 1.5× interquartile range; bounds of box, minima, maxima, and black points represent discrete values. The *N* represents the number of measurements in each comparison. The proteins with at least 50% measurements in replicate groups are selected. The red lines represent theoretical log2 fold change. The root mean square error (RMSE) values are calculated. Source data are provided in the supplementary data file.

The precision is evaluated by comparing the coefficient of variation (CV) of each method, as shown in **Figure 9**. The proteins with at least 50% measurements in replicate groups are shown. The quantms with MSstatsTMT achieved the lowest CVs median, and the CVs are significantly reduced by MSstats/MSstatsTMT than quantms with summed intensities. CV median is comparable between quantms and MaxQuant. In addition, the TMT method greatly improves the accuracy of protein quantification.

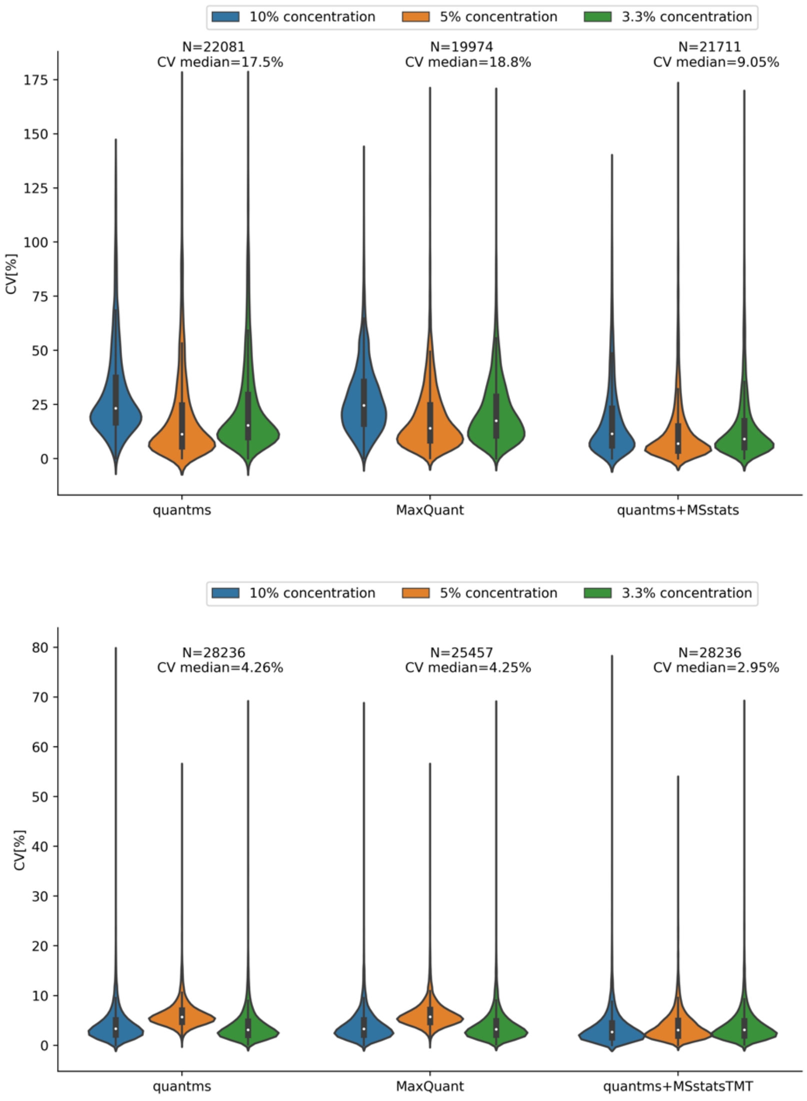

Figure 9: Violin plots of the distribution of coefficient of variations for quantms with summed protein intensity, MaxQuant raw intensity and quantms with MSstats/MSstatsTMT aggregation methods on label-free (top panel) and TMT (bottom panel) experiments, relatively. The proteins with at least 50% measurements in replicate groups are only considered. Box, median ± interquartile range; whiskers, 1.5× interquartile range; bounds of box, minima, maxima. The N represents the number of measurements. Source data are provided in the supplementary data file.

Supplementary Note 4: Large-scale datasets analysis

Previous efforts from the PRIDE team used MaxQuant in a big-memory node (60 CPUS, 300 GB memory) to reanalyze multiple datasets to compute IBAQ values [9]. We compare the runtime of those reanalyses with quantms runtime in an HPC cluster with 204 compute nodes including 10 nodes of more than 500 GB memory. While the comparison between a single node execution and multiple node cluster is difficult to perform, we highlight in **Figure 10**, the runtime for Ananth *et. al.* [9] analysis compared with quantms analysis (only LFQ datasets).

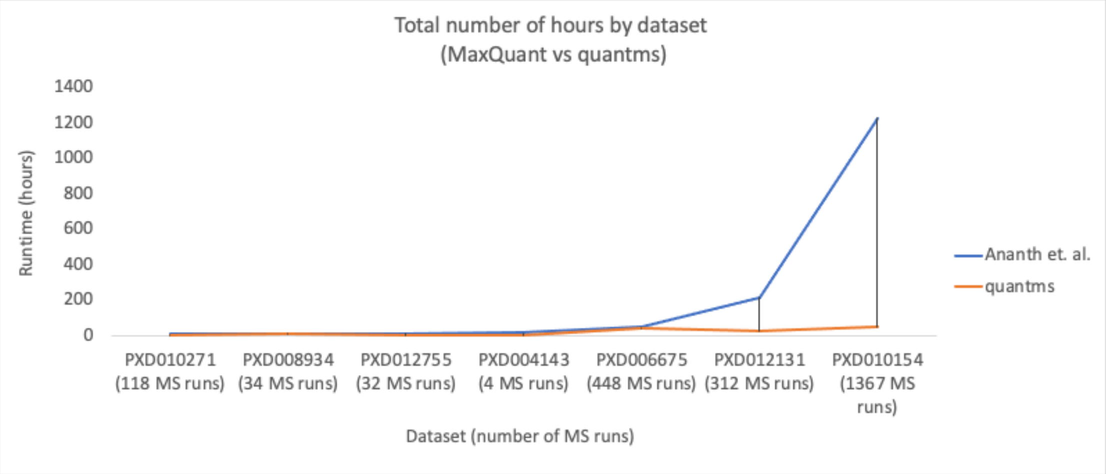

Figure 10: Runtime comparison between quantms and Ananth et. al. (MaxQuant) reanalysis.

No major differences are observed when analysing small datasets (less than 100 MS runs). However, when the number of MS runs and samples grows the runtime differences increase. quantms benefits for the parallelization and distribution of MS runs in some of the processing steps (peptide search, percolator, multiple search engine merge), decreasing the time to process big submissions.

Supplementary Note 5: DIA benchmark datasets.

PXD026600: DIA analysis of the UPS1 E. coli proteomic standard.

ProteomeXchange dataset PXD026600 [22] is a proteomic standard composed of 48 human proteins with different concentrations (a commercial mixture called UPS1, Sigma-Aldrich) spiked in a whole E. coli protein extract background. Data is obtained at 8 UPS concentrations on an Orbitrap Fusion instrument with 4 different DIA window schemes (narrow, wide, mixed, and overlapped). In this benchmark, all raw files were analysed by the DIA workflow of quantms (DIA-NN library-free parallelization). **Figure 11** shows the ability of the workflows to quantify proteins in a complex biological sample and to detect species present at low concentrations by reporting the number of E. coli and UPS1 quantified in each of the 8 concentrations. The workflow achieved nearly perfect performance (quantified all 48 UPS proteins) at 4 high concentrations (**Figure 11B**).

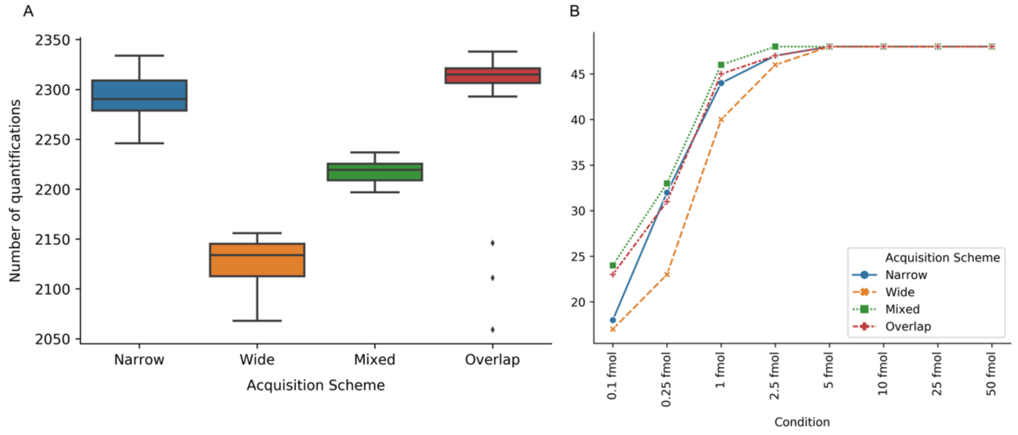

Figure 11: The number of E. coli (A) or UPS1 (B) was quantified in the three replicates of each sample. Box, median ± interquartile range; whiskers, 1.5× interquartile range; bounds of box, minima, maxima, and black points represent discrete values in boxplots.

Figure 12 shows the log2 intensities of all proteins quantified by quantms and MSstats. The log2 intensities appear consistent in all replications for each acquisition scheme. For UPS1 spiked proteins, the log2 intensities are more consistent with increasing concentration. At low concentrations, considering the presence of more noise signals.

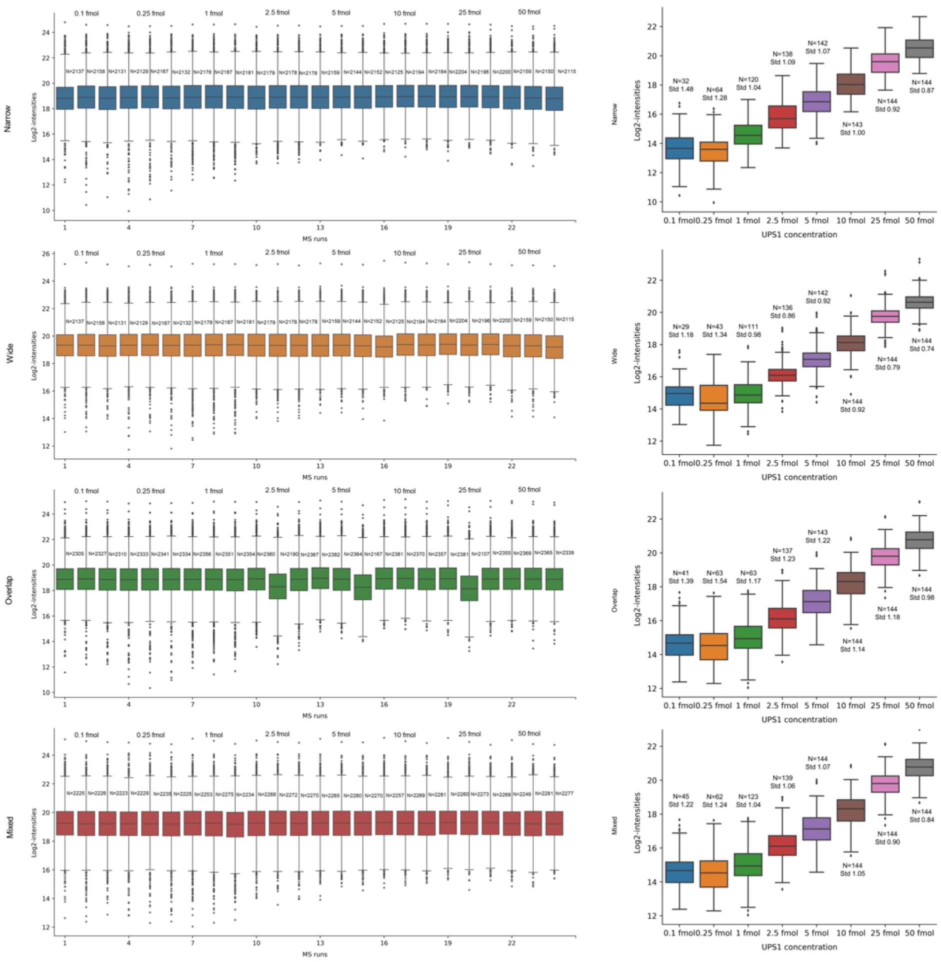

Figure 12: (left) Distribution of total protein log2 intensities obtained using quantms with MSstats in each ms runs. The *n* represents the number of proteins quantified (right) Boxplots of UPS1 protein intensity distribution at each UPS1 concentration. The number and standard deviation of measurements are denoted by *n* and *std*, respectively. Box, median ± interquartile range; whiskers, 1.5× interquartile range; bounds of box, minima, maxima. The black points represent all discreet values. Source data are provided in the supplementary data file***.***

To evaluate the technical reproducibility and consistency, we calculate a coefficient of variation (CV) of protein intensities in triplicate concentrations (**Figure 13**). We considered only precursors with intensity values without missing values at each UPS1 concentration. For E. coli background proteins, all have an average CV below 10% except for the Overlap (21.6 % CV – Similar results were found in the original manuscript [22]), affirming the high reproducibility of DIA analysis for label-free quantification. For UPS1 proteins, the CV was better for UPS1 proteins at high concentrations averaging respectively 3.85% at 50fmol/µg. But The Overlapped windows scheme dataset had higher CVs respectively 48.42%, 43.90% and 42.73% at 2.5, 5 and 25fmol/µg (**Figure 13B**). Such discrepancies in the CVs might be due to the overlapped scheme dataset having technical issues during the acquisition of 3 DIA files for one replicate each at 2.5, 5 and 25 fmol/µg.

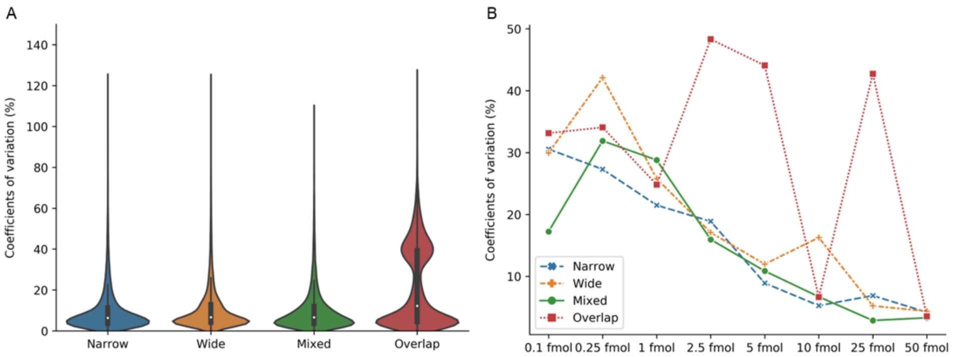

Figure 13: Coefficient of variation of E. coli (A) or UPS1 (B) proteins based on the 3 replicates with each acquisition. Source data are provided in the supplementary data file. Box, median ± interquartile range; whiskers, 1.5× interquartile range; bounds of box, minima, maxima in figure A. We considered only precursors with intensity values without missing values at each UPS1 concentration.

We calculated the Fold Change (FC= log2(ratio) of protein intensities for each possible pair comparison between concentration conditions. The quantification results are processed and analysed by MSstats. Plotting the mean absolute percentage error (MAPE) for each expected FC (**Figure 14**) reveals an overall tendency for the error to increase when a very low UPS1 concentration is compared to a high concentration. On the other hand, the MAPE decrease as the absolute difference decrease between the two compared concentrations. The error over all workflows was <10% when real concentrations above 1 fmol were compared.

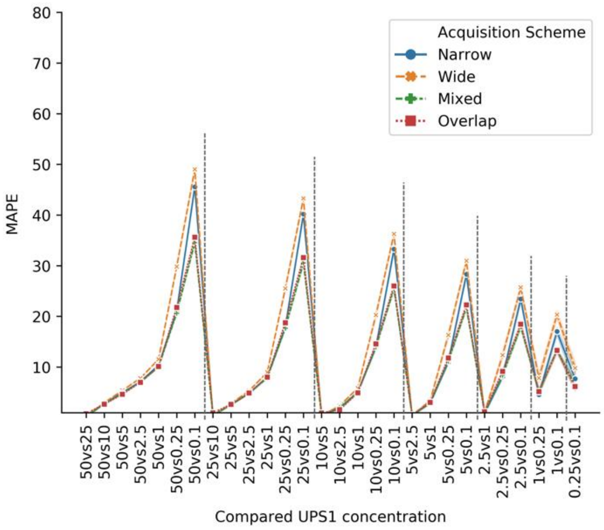

Figure 14: Mean absolute percentage error of detected spiked-in proteins concentrations relative to the corresponding known concentrations for each acquisition scheme in the PXD026600.

We assess the impact of acquisition schemes and concentrations on the estimation of differentially expressed proteins using receiver operating characteristic (ROC) curves of the adjusted p-values associated with protein fold changes and by reporting the corresponding areas under the curve (AUC) based on known differentially expressed proteins (UPS1). In most of the conditions, the workflow achieved a perfect distinction between the two classes compared, namely UPS1 proteins (differentially expressed) and E. coli proteins (fixed background) (**Figure 15**). We observe a high AUC of on average 0.994 for high concentrations (at least one of the compared UPS1 concentrations ≥ 5 fmol/µg) that drops to 0.802 for low concentrations (both ≤ 2.5 fmol/µg). This is likely due to less identification and less accurate quantification of UPS1 proteins at lower concentrations.

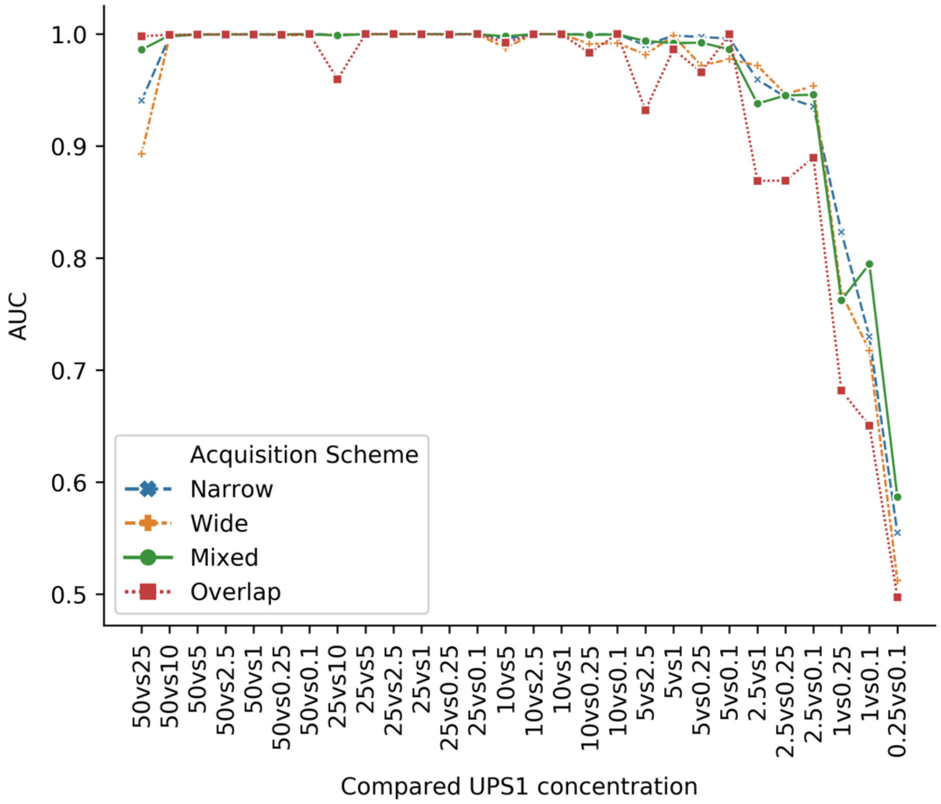

Figure 15: The area under the curve (AUC) corresponding to receiver operating characteristic (ROC) curves on adjusted p-values plotted for 28 pairwise comparisons of two UPS1 concentrations for each acquisition scheme.

Supplementary Note 6: Single cell benchmark datasets.

PXD016921: The single-cell dataset consists of 7 samples comprising zero, 1, 20 and 100 cells. Identifications in blank control samples serve as false-positive identification. All raw files were processed using quantms for feature detection, database searching, and protein/peptide quantification. The MBR is enabled, and both peptides and proteins were filtered with a maximum false discovery rate (FDR) of 0.01. The modifications and enzymes are the same as the original research. Other unmentioned parameters are the quantms default settings. Overall, 12269 unique peptides and 1903 protein groups are quantified, an increase of 6% compared with the original results. Moreover, only 43 false positive proteins are quantified in the blank sample, a decrease of 50% compared to reference results. Accordingly, quantms discard more noise and false-positive identifications and match more confident features.

Table 6: Number of unique peptides and protein groups identified from analysis of 100 HeLa cells, 20 HeLa cells, four single HeLa cells (SC-1, SC-2, SC-3, SC-4), and zero cells (blank) using quantms and MaxQuant with MBR identifications.

	100 Hela cells	20 Hela cells	Single cell 1	Single cell 2	Single cell 3	Single cell 4 (Large Hela cell)	Blank
quantms							
Unique peptides	10858	8237	3604	2871	2900	4344	43
Protein groups	1831	1583	1062	883	705	1052	43
MaxQuant							
Unique peptides	9343	7462	3380	3395	2772	5504	95
Protein groups	1658	1472	904	928	773	1286	87

PXD023366: In addition, we also collected and analyzed three biological-oriented single-cell experimental datasets. PMID[35809850] published a single-cell quantitative proteomic dataset of human Oocyte maturation, which involved three condition groups: germinal vesicle (GV) stage, oocyte in vitro maturation (IVM) and

oocyte in vivo maturation (IVO). Twelve biological replicates of each type were analyzed using quantms with similar parameters as the original paper.

quantms workflow quantified 25% (601) more total proteins and 20% (4130) total peptides than the original paper. Of these, 2182 proteins with at least two unique peptides are expressed in at least 50% of samples in at least one group, and more than 70% total number of proteins are quantified in each oocyte sample. The differential protein expression analysis was performed using MSstats including equalizeMedians normalization, Tukey's median polish estimation method and imputation parameters. Only proteins with fold change >1.5 and FDR q < 0.05 were considered significant between groups. We compared our data for proteins with differential expression levels to the reported transcriptome profile data and original paper. Overall, 198 differently expressed proteins (DEPs) are detected. Moreover, of the 136 novel DEPs reported in our results, 55 differential expressed proteins were also detected as differentially expressed genes in independent transcriptome studies (Figure 16A). To evaluate the pathways regulated during oocyte maturation, KEGG pathways were analyzed (Figure 16B). Both oxidative phosphorylation and ribosomal pathways were detected in comparison to the initial study. Novel DEPs reported in quantms, and mRNA levels are also enriched to Amyotrophic lateral sclerosis novel pathways. It would indicate that quantms results can provide additional biological and biomedical insight.

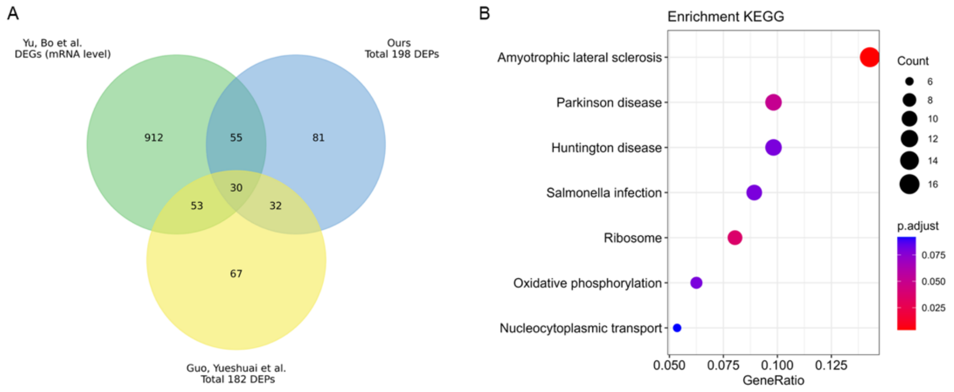

Figure 16: (A) Venn diagram of comparison of genes differentially expressed at the proteome (quantms and original results) and transcriptome levels. (B) Dot plot of enriched KEGG pathway terms in differentially expressed proteins between GV and IVO oocytes. Unadjusted P values were calculated by a one-sided hypergeometric test. Then P values are adjusted by the Benjamini-Hochberg method. KEGG terms with adjusted P < 0.1 are shown.

The Figure 17 indicates high reproducibility of protein quantification, with pairwise Pearson's correlation coefficients medina 0.863, 0.847 to 0.893, and 0.893 among oocytes in GV, IVM, and IVO group, respectively. Furthermore, the PCA analysis showed that closer clustered patterns in GV and IVO groups than IVM group, which appeared as a distinct group (Figure 18).

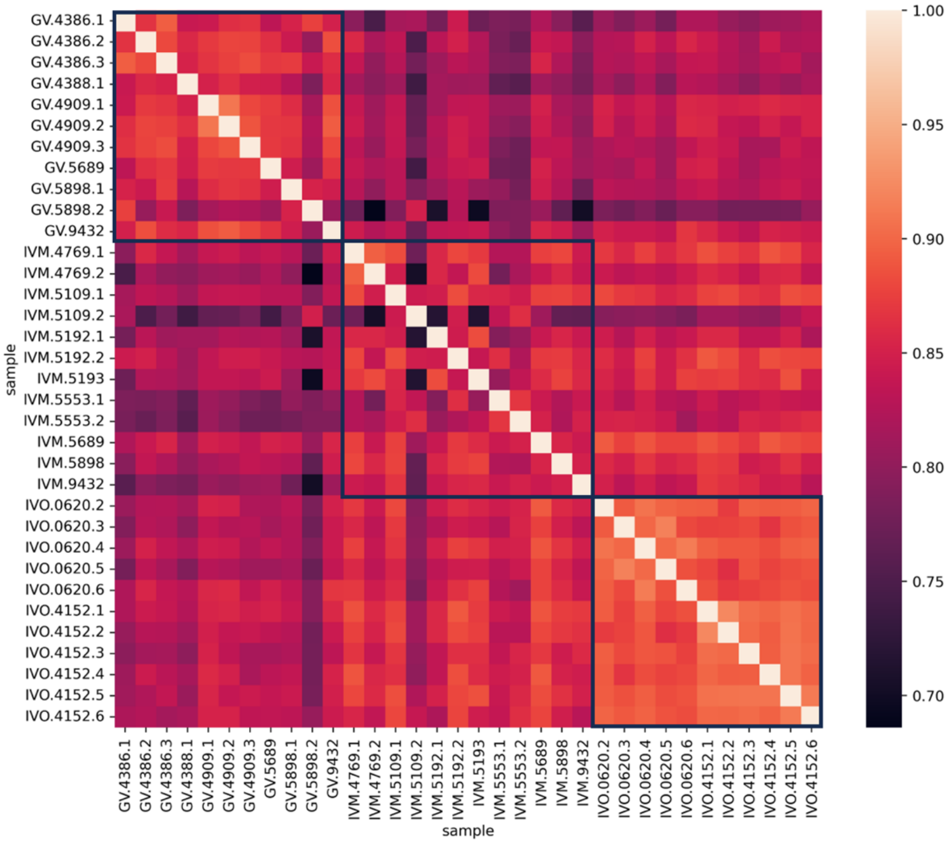

Figure 17: Heatmap of pairwise correlation analysis was conducted on 34 oocytes, using log2 intensity values by MSstats. Black boxes were used to identify oocytes from each group.

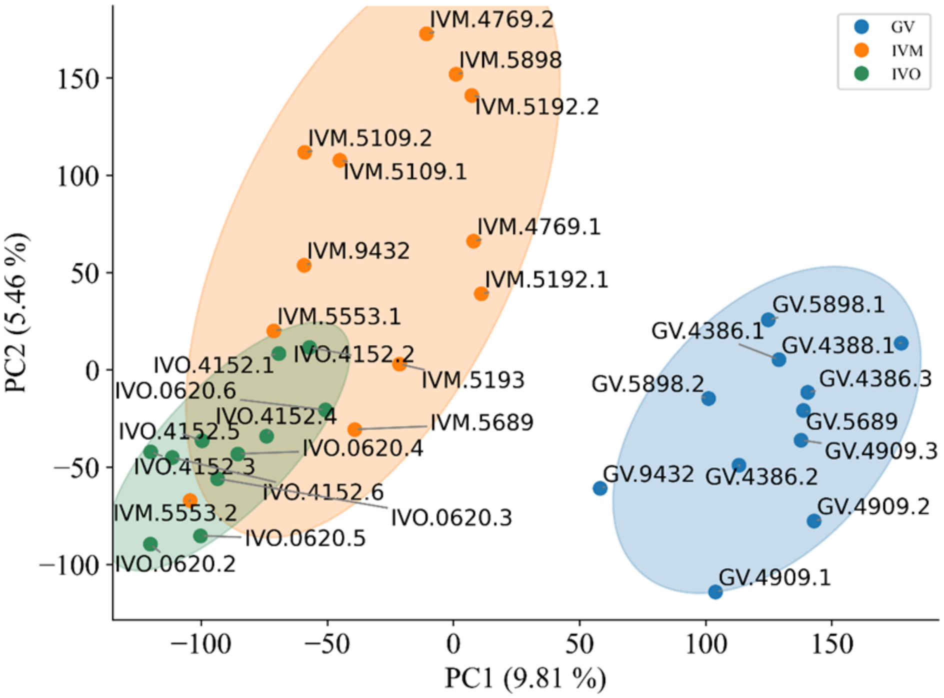

Figure 18: PCA of the proteomic data from 34 human oocytes with different colors representing the sample group.

PXD024043: We have re-analyzed the cell cycle dataset that has frequently been used as a test case in single-cell studies. A total of 3171 proteins are quantified using DIA-NN with a library-free model, resulting in a 26% increase in quantified proteins compared to the original article. This number ranges from a median of 1076 in G1 to 2217 in G1/S, 1709 in G2, and 1958 in G2/M (Figure 19). Then the peptide intensities are processed and aggregated by MSstats with equalizeMedians normalization and TMP summary methods. The PCA of proteomes shows that different clustered patterns among four cell cycle stages, in agreement with the original research (**Figure 20**). In addition, we also re-analyzed the diaPASEF datasets with five diaPASEF scan repetitions using the workflows above and quantified 7% more proteins and 18% more peptides with smaller proportion of missing values than the reference results, although there was a minor loss in quantitative reproducibility (**Table 7**).

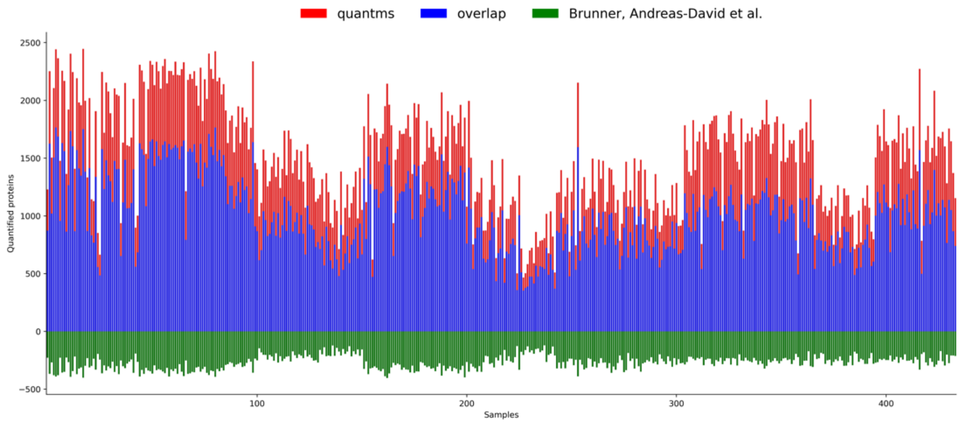

Figure 19: Numbers of protein quantifications across 434 cells in the indicated cell cycle stages. Proteins quantified only in quantms are red, overlaps are blue and only in the original paper are green.

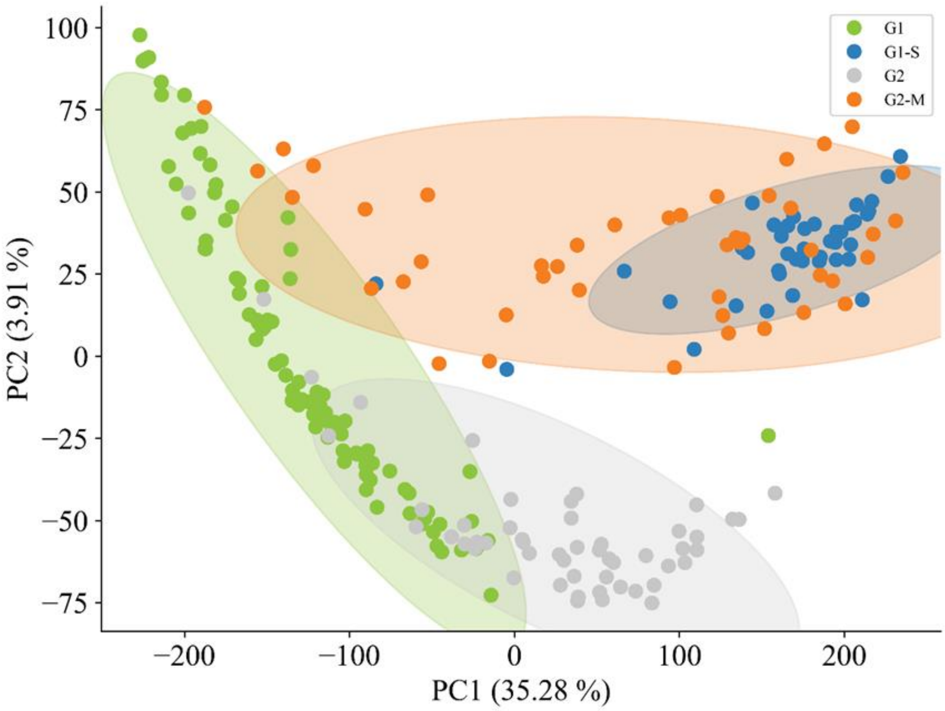

Figure 20: PCA of single-cell proteomes across 231 cells including four cell cycle stages.

Table 7: Comparison of proteins, peptides quantified, median coefficient of variation and missing values in the PXD024043 diaPASEF dataset.

diaPASEF scan repetitions	**1**	**2**	**3**	**4**	**5**
quantms					
peptides quantified	4425	4451	4470	4464	4452
proteins quantified	23540	23678	23895	23770	23848
median coefficient of variation [%]	9.65	10.45	6.99	15.10	8.99
missing values [%]	5.6	5.8	3.7	7.1	4.5
Reference					
peptides quantified	4204	4180	4251	4166	4205
proteins quantified	20276	20430	20993	20005	20407
median coefficient of variation [%]	9.92	8.80	5.84	12.34	6.11
missing values [%]	6.2	7.1	4.8	10.7	6.9

PXD023904: The U2OS single-cell datasets are collected and reanalyzed using quantms with MSstats. Original research reported six distinct nuclei classes, with class 1 representing mitotic states and the remaining five nuclei classes representing interphase with varying feature weighting. We quantified up to 4459 protein groups from five nuclei classes by DIA-NN sub-workflow with library free mode, an increase of 14.5% over the original results, and there are 73 proteins that quantified only in quantms are considered as high abundance (top5%). To further verify and explore performance of single cell analysis, principal component analysis (PCA) is performed, classes 4 and 6 are relatively discrete groups, but the more frequent classes (2, 3 and 5) grouped together (**Figure 21**).

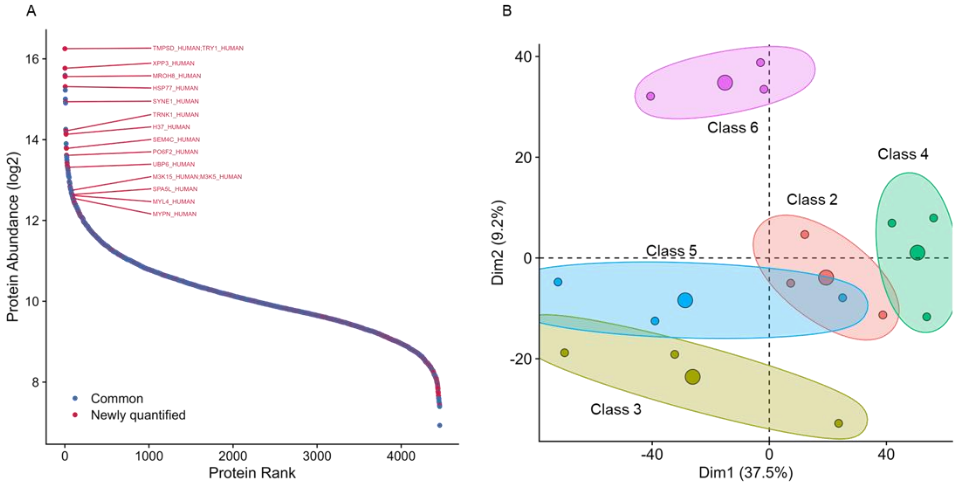

Figure 21: (A): Protein rank from quantms with MSstats. (B) PCA of single-cell proteomes across 231 cells from six distinct nuclei classes.

Supplementary Note 7: Data reanalysed with quantms.

A total of 118 datasets were reanalyzed using quantms version 1.1, UniProt protein sequence database (version 10.20222), only the SwissProt (reviewed proteins) without isoforms information based on specific dataset selection, peptide identification, quantification and quality control rules (Table 10). All datasets have been deposited in PRIDE Archive public FTP:

- Differential expression datasets (35): http://ftp.pride.ebi.ac.uk/pub/databases/pride/resources/proteomes/differential-expression/
- Absolute IBAQ-based datasets (83): http://ftp.pride.ebi.ac.uk/pub/databases/pride/resources/proteomes/absolute-expression/

All datasets were filtered at a 1% false discovery rate (FDR) at PSM and protein levels for all datasets. All parameters including the posttranslational modifications, precursor and fragment tolerances can be found for each dataset in the corresponding SDRF [23]. Table 8 shows the list of datasets reanalyzed including the number of samples, ms runs, peptide spectrum matches, proteins quantified and total runtime for each dataset.

Then PXD030881 are selected to demonstrate that the majority of differential proteins only found by quantms reanalysis are supported by differential genes from two transcriptomic studies. Statistical downstream analysis with the MSstatsTMT (integrated into quantms) tool detected 3381 (original) and 4301 (reanalysis) differential proteins (DEPs, adj. p-value < 0.05) [PMID 35335125]. We compared the 1762 DEPs found only by quantms with two independent transcriptomics results [PMID23077249, PMID34395436]. 1035 of the 1762 novel DEPs reported in our results were also detected as differentially expressed genes in the two independent transcriptome studies.

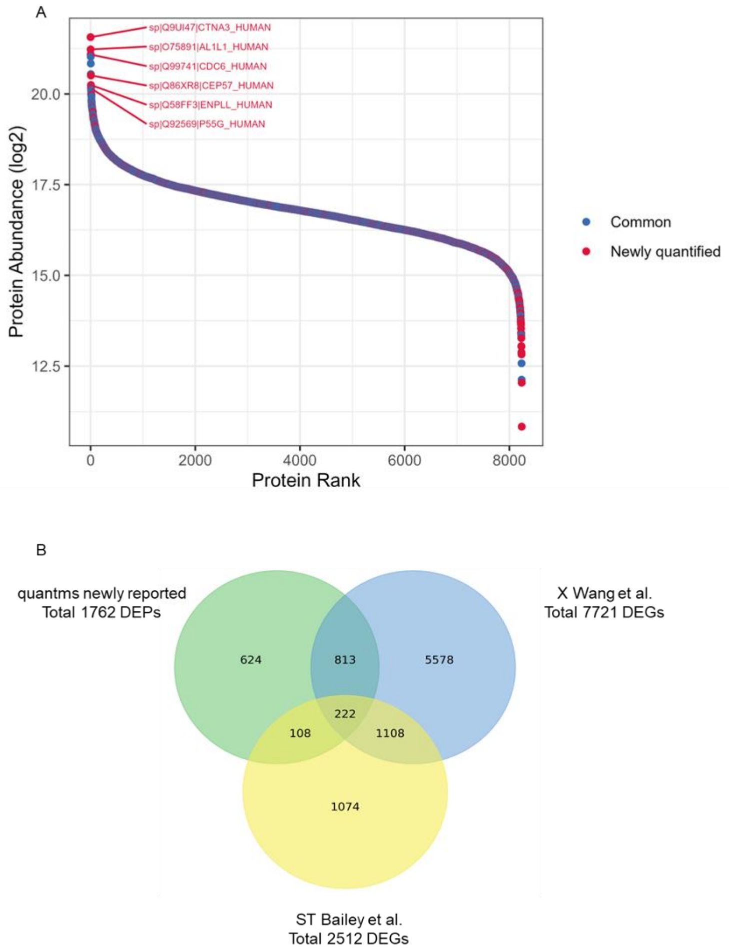

Figure 22: (A) Protein rank plot from quantms, and the protein quantified by only quantms are marked red. (B) Comparison of differential proteins reported only in quantms compared to the original research with those reported in two transcriptome studies.

Table 8: Datasets reanalyzed using quantms, (DE) differential expressed studies, (AE) intensity-based absolute expression studies.

Accession	*Type*	*# msruns*	*# samples*	*# of MS*	*# PSMs*	*# peptides*	*# proteins quantified*	*# original peptides*	*# original proteins quantified*	*runtime*	*cpu runtime (hours)*
PXD004683	DE	48	12	1197561	242962	64235	4903	50754	5513	05:32:32	45
PXD004684	DE	15	8	801646	340501	30074	3503	12976	2155	01:10:44	3
PXD004873	DE	76	38	6336044	1788264	31725	3404	11787	2579	03:20:35	43
PXD012574	DE	24	30	1053235	235755	47577	4867	20868	4905	03:54:32	35
PXD022992	DE	12	6	1056747	1491915	135706	9474		8455	06:14:59	17
PXD023423	DE	36	6	1531343	487859	94888	6683			08:06:50	54
PXD025560	DE	203	203	28625259	5413288	88199	8523		6717	35:10:31	181
PXD030881	DE	24	10	1684376	407792	116306	8260	84448	7569	09:53:45	44
PXD032263	DE	8	4	684223	933494	112288	8079	77928	7089	04:51:56	10
PXD033169	DE	50	50	4124332	280236	5190	548			02:11:06	32
PXD027817	DE	48	48	4319862	138587	2373	329			00:55:42	24
PXD002137	DE	192	32	14938434	5189483	166811	10550	111680	9112	63:06:12	265
PXD003497	DE	60	30	4677480	1231053	22914	2439			00:58:26	127
PXD025864	DE	18	6	888111	318986	31954	3101	26657	2877	01:39:17	1.7
PXD023508	DE	80	80	23183808	157303	2805	265		380	15:13:52	250
PXD003539	DE	120	60	9385023	1647895	23413	3509	18030	2174	32:48:50	176
PXD023508	DE	80	80	23183808	157373	2805	265		380	18:22:56	250
PXD018830	DE	25	25	1422778	963529	64270	6333	28746	4617	03:19:41	12
PXD021394	DE	24	24	1550158	1123224	63072	6577		4644	02:55:21	13
PXD028618	DE	18	18	3636018	295437	26261	3478		1414	03:15:37	16
PXD010429	DE	4176	348	27150819	2887185	162572	8227	158160	8300	19:42:00	553

PDC000126	DE	204	170	10470310	1343724	175954	9638			20:42:10	131
PXD002395	DE	198	33	7779229	4765530	181082	10530	126235	9307	12:02:07	211
PXD000672	DE	36	36	2610072	726081	25636	2971		1632	12:47:01	173
PXD004691	DE	224	97	13851936	3709841	26493	2956	17108	2009	05:39:07	181
PXD014943	DE	113	113	15031034	4406765	57115	5609	31952	4365	15:59:52	176
PXD028251	DE	51	51	4444942	1366811	7134	538		548	16:09:47	54
PXD014414	DE	24	30	1733512	101040	21693	3130			10:14:45	10
PXD014145	DE	12	11	390692	146489	79960	6482			02:36:30	9
PXD020248	DE	4	12	215854	100786	34042	3458		3500	02:32:31	4
PXD020109	DE	12	4	719423	80722	7736	1217	6366	1173	01:35:02	22
PXD030671	DE	18	33	742449	184438	64775	4665		4051	01:36:06	22
PXD027008	DE	10	10	3359586	509878	37299	5282			05:54:51	34
TOTAL DE (35)		**6’560**	**1’916**	**222’780’104**	**46’488’978**	**432’134**	**14’439**				**3’178**
MSV000079033.1	AE	6	30	1049462	151881	20836	1640			01:57:06	51
MSV000079033.2	AE	10	30	971658	156354	21666	1635			01:21:14	29
MSV000079033.3	AE	4	30	1123122	221228	22692	1634			01:40:09	38
MSV000079033.4	AE	16	120	4435487	1035877	48272	2937			0:21:57	91
MSV000079033.5	AE	12	89	3364789	625221	25288	1691			01:47:18	76
MSV000087095.1	AE	50	50	6714014	929619	25993	2755			02:17:00	28
MSV000087095.2	AE	92	247	29391794	4170441	90242	6108			08:26:25	367
PXD000561	AE	2211	85	31432856	9539188	289928	12877			10:18:00	1040

PXD000865	AE	1055	34	11849511	4584514	171870	8974			04:30:05	233
PXD000612	AE	231	11	16822603	6045925	144138	8814			14:34:24	604
PXD002179	AE	3	148	4188548	2335549	151174	8823			02:11:53	48
PXD002854.1	AE	73	2	1117906	196797	4306	332			1:24:20	15
PXD002854.2	AE	17	6	263386	53985	4836	493			0:37:56	3
PXD002854.3	AE	84	22	1289544	231699	3808	267			18:43:11	41
PXD006559	AE	10	72	1103416	210748	71736	5160		5816	00:32:29	20
PXD010154	AE	1367	38	76331811	13644346	344215	14602	277698	13413	49:30:00	6467
PXD020192	AE	92	46	14082788	1445072	59994	6187			13:27:08	328
PXD010271	AE	118	117	4144357	655277	69667	5442	68623		00:46:17	92
PXD004452	AE	184	4	4525141	347827	115974	8693			00:50:59	122
PXD016999	AE	336	280	33132420	3312949	185984	10322		10442	09:40:21	762
PXD016999.1	AE	336	280	30051951	3409423	187453	10351			07:29:58	116
PMID24274931	AE	80	4	1024500	102355	16381	1814			01:32:00	15
PXD012755	AE	32	32	1842536	816445	20519	2325	8017	1640	00:36:11	14
PXD004143	AE	4	2	218281	40582	19282	3518			02:15:57	6
PXD005445	AE	105	7	6657738	2816862	66690	9220		8980	06:17:38	81
PXD005445.1	AE	77	77	5563556	2067956	151812	5205			03:57:49	62
PXD008441	AE	115	115	2752279	378840	20287	1658		1929	06:47:37	46
PXD008467	AE	100	304	2431586	92813	6813	571			00:35:24	41
PXD008468	AE	710	709	5126445	229568	7215	571			01:56:39	92
PXD009219	AE	30	127	583805	107639	2103	117			02:14:13	21
PXD012131	AE	312	26	50179092	4575069	166338	10231	129050	9735	25:45:04	459
PXD019909	AE	154	12	17118239	3223352	243137	11680	173228	10701	12:40:26	350
PXD019909.1	AE	43	43	3393681	2444015	127893	9445		9140	07:29:58	15

PXD008934	AE	34	34	3637030	1133506	42867	3433	31088	2933	11:11:00	54
PXD006675	AE	448	56	39666383	10788990	210623	11178		11163	39:58:57	2082
PXD008722	AE	252	21	9250425	3397589	134566	6851	107417	6436	10:04:27	239
PXD018678	AE	46	46	2651540	2013963	35387	3947			02:52:05	27
PXD018678.1	AE	12	1	881877	262936	67611	5930			02:16:35	14
PXD012636	AE	90	9	3989225	1725188	113962	6425	110736	6243	09:31:27	120
PXD011349	AE	55	55	1093212	344925	12061	1309		1445	01:26:30	12
PXD005736	AE	24	2	1728473	387828	123784	8518			02:34:57	27
PXD008840	AE	504	84	36907863	6493137	147323	9296		9186	44:30:24	1094
PXD022661	AE	60	5	667548	159833	12788	1667	12096	1492	00:48:54	11
PXD013523	AE	96	16	4253344	1657534	65554	5855	85768	7414	05:36:26	58
PXD019123	AE	27	9	1281714	423137	24714	2860	24636	3180	02:04:54	16
PXD030304	AE	6862	2013	1135197814	241592436	118322	8941		8498	25:10:24	23352
PXD003947	AE	108	10	4063798	1003593	84584	5087		4727	7:58:45	46
PXD004242	AE	1290	58	30415188	4562256	8029	529		448	30:44:08	1363
PXD008333	AE	201	8	7804654	1993476	98165	8144	83984	7609	11:28:47	122
PXD009348	AE	168	7	4589074	375058	19466	1707	14588	1826	4:03:43	64
PXD009737.1	AE	36	1	2249213	570500	153489	10477	257785	10743	2:52:17	27
PXD009737.2	AE	36	1	2051259	356753	98641	7839	247234	8849	1:54:42	19
PXD009737.3	AE	36	1	1912707	433716	91533	8439	253038	9700	2:09:58	18
PXD010899.1	AE	1808	26	26814390	1391181	41042	3288	15424	1626	24:41:47	714
PXD010899.2	AE	282	40	5881201	172728	16497	1506		1080	1:33:03	448
PXD0011839	AE	398	84	-	1082541	20683	1773		2081	39:52:41	-
PXD013231.1	AE	105	7	2850320	339615	8874	552	8641	661	3:40:08	38
PXD013231.2	AE	1549	1549	48873931	9759381	7437	562		465	43:13:51	931

PXD017052.1	AE	114	38	6465743	1062715	14549	1293			1:58:04	69
PXD017052.2	AE	3139	3140	265250164	41579239	29694	3230			119:35:0	6121
PXD017834.1	AE	9	3	808262	24665	2716	172	2332	241	2:47:25	9
PXD017834.2	AE	18	6	1311243	71847	5004	305		376	1:35:51	20
PXD019817	AE	98	98	2731283	197079	3469	242		199	4:04:36	60
PXD020727	AE	41	41	2980280	126333	7398	657		598	01:38:54	29
PXD022469	AE	384	16	5327717	630420	18239	924			6:28:40	188
PXD023650	AE	48	1	916155	97510	29383	2797	19508	2190	1:14:54	24
PXD024364.1	AE	281	9	25860113	7249804	469414	14615	396782		11:06:10	2542
PXD024364.2	AE	366	14	2639921	1236716	143556	10466	152259		2:52:41	205
PXD027125	AE	45	45	6037471	187203	3015	330			04:48:08	19
PXD029009	AE	366	366	4812900	690169	1901	161		321	02:37:43	301
PXD030598	AE	435	870	214304800	2847574	3827	385		366	06:57:53	1753
PXD032212	AE	18	9	1378389	540117	37557	3138		1496	04:53:20	52
PXD034244	AE	29	9	3855168	1088315	40832	5064		4659	02:59:18	28
PXD036609	AE	27	27	2639921	168495	8919	944			4:39:18	55
PXD037340.1	AE	18	6	451227	129986	15276	1445			01:17:39	8
PXD037340.2	AE	110	31	3079929	1006539	36550	3142			01:07:08	16
PXD037682	AE	60	60	2400000	156312	2113	170			04:40:28	70
PXD038526	AE	50	50	2230000	126005	2103	158			01:25:15	64
PXD038669.1	AE	9	1	212189	35744	2288	183			00:25:15	3
PXD038669.2	AE	144	31	2129011	493916	4211	374			01:05:35	25
PXD038674	AE	48	16	616997	219695	13871	1230		1429	02:05:47	13
PXD039023	AE	879	879	11812679	2422620	2620	214			8:10:39	1720
PXD040438	AE	24	24	1293983	96783	5759	383		447	02:54:43	17

TOTAL AB (83)	**29354**	**13132**	**2’324’536’ 030**	**425107017**	**1037817**	**17463**	**40’045 (1.4 / ms run)**

Table 9: The table of rules to select datasets and statistical framework including identification rules, quantification rules and quality control rules.

Dataset selection rules	
Rule	*Comments*
Dataset publication.	*All datasets must be previously published in a scientific journal.*
TMT, iTRAQ, DDA-LFQ, DIA-LFQ	*Datasets were generated using one of the following analytical methods. quantms team has performed multiple studies to evaluate how intensity-based quantitation from plex, LFQ and DIA studies are comparable using quantms. References: [10.22541/au.168174437.77664121/v1, 10.1021/acs.jproteome.2c00812].*
The following information was manually reviewed and annotated based on the original manuscript for each dataset:<br>- *Instrument used.*<br>- *Artefactual Posttranslational modifications or enrichment performed for the sample.*<br>- *Tissue information.*<br>- *Cell type.*<br>- *Disease state: In this case we manually annotated for all experiments the healthy samples.*<br>- *Sample relation to files: All samples were correctly annotated including replicate information (biological/technical), fractions associated with each sample.*<br>- *Precursor and fragment tolerances: All precursor and fragment tolerances are annotated manually into an SDRF. The values were extracted from the original manuscript and refined using the following tool: param-medic [10.1021/acs.jproteome.7b00028]*	*All datasets are annotated in SDRF and can be found in the following repository in GitHub: https://github.com/multiomics/multiomics-configs/tree/master/projects/tissues quantms uses these annotations automatically to set the settings for each file and each tool in each dataset appropriately*
Peptide and protein identification rules	
Rule	*Comments*
Protein database: Uniprot-Swissprot Reviewed database without isoforms.	*We selected the Uniprot-Swissprot reference database to decrease the detection of features that can be reproduced across samples and*

	decrease the impact in quantitation results of the shared peptides. We aim with this reanalysis's reproducible quant values across samples instead of more protein identifications.
Enzyme used: Trypsin	*We reanalyzed only datasets which use trypsin as the enzyme (cleavage agent). As the previous rule, we aim to have features that are more reducible across samples and datasets. While the number of datasets from other enzymes are growing in the public domain, most datasets are based on Trypsin. This decision helps to get consistent iBAQ values for all datasets because the peptide spaces (tryptic space) are the same.*
Dataset level PSM and Protein FDR: 1%	*By applying a strict 1% FDR at PSM-level (before quantification) and protein(-group) level on a final dataset-wide scale (after quantification) we guarantee the statistical control of the FDR for each dataset.*
Peptide Length	*We filter out all peptides less than 7 AA. This filter can be applied using the ibaqpy library from quantms framework (https://github.com/bigbio/ibaqpy). Future resources or views of the same data can apply more stringent filters, for example AA length > 9.*
Number of unique peptides per proteins >=2	*For a protein to be considered as reliably quantified, at least 2 unique peptides were needed.*
Quantification rules	
Rule	*Comments*
Number of samples per feature.	*Every feature (Peptide + charge + retention time + replicate) must be present in at least 20% of the samples of each dataset.*
Number of projects per protein and global FDR	*We have a multi view approach for all the proteins quantified:*<br>*- Dataset View: In this representation FDR is controlled at the level of the dataset as explained in previous section (1%).*<br>*- Tissue proteomes View (https://quantms.org/baseline): To achieve this, we began by merging all protein lists with protein q-values from various datasets, while keeping them separated based on the dataset type (e.g. cell lines and tissue). Next, the algorithm generated a distribution of decoy proteins that were similar to the*

	target proteins in the integration list. The protein-adjusted FDR was calculated based on this distribution of decoy proteins. Finally, we applied a strict protein-adjusted FDR threshold of less than 0.01 to filter the integration results.

Data provenance and QC

Rule	*Comments*
All results must be in standard file formats as follows:<br>- *Sample metadata: SDRF*<br>- *Spectra: mzML*<br>*Peptide/Protein identifications: mzTab*	*All the results from these reanalyses are in standard file formats.*
Peptide, Protein: scores	*All peptide and protein scores including posterior error probabilities, p-values and search engine scores are available.*<br><br>*This can help the community and future resources to detect manually low-quality signals (peptides and protein identifications/quantitation values).*
pmultiqc (https://github.com/bigbio/pmultiqc)	*Quality control reports for every dataset are provided using the newly developed library pmultiqc (https://github.com/bigbio/pmultiqc). The library well explained in the manuscript and the quantms documentation provides multiple plots and statistics to detect and visualize problems in the quantitative results.*

Table 10: The number of proteins quantified with at least two unique peptides after global adjusted FDR in different tissues and cell lines from AE experiments.

Source	Number of proteins quantified
liver	14203
testis	14044
lung	13940
stomach	13780
brain	13533
colon	13528
heart	13470
placenta	13434
pituitary hypophysis	13343
adrenal gland	13256
ovary	13224
pancreas	13201
small intestine	13128
prostate	13009
lymph node	12961
thyroid gland	12937
spleen	12927
fallopian tube	12873
duodenum	12853
kidney	12796
urinary bladder	12763
esophagus	12712
salivary gland	12709
gallbladder	12675
uterine endometrium	12621
rectum	12609
bone marrow	12511
appendix	12460
smooth muscle	12368
adipose	12269
skin	11857

cell lines	11374
spermatozoon	8269
retina	8049
frontal cortex	7600
cd8 tcells	7459
b cells	6999
cerebellum	6597
spinal cord	6574
tonsil	6545
gut	6515
breast	6348
cd4 tcells	6309
monocyte	6246
nk cells	6242
uterus	6234
seminal vesicle	6109
cerebral cortex	5819
blood plasma	4993
oral epithelium	5643
tube	5558
platelet	5084
uterine cervix	5003
cardia	4921
uterus_pre-menopause	4874
endometrium	4836
platelets	4706
epidymis	4596
temporal lobe	4585
occipital cortex	4548
parietal	4508
ascites	4400
uterus_post-menopause	4305
medulla oblongata	4113
bladder	3824
trachea	3793
anus	3744

ureter	3732
earwax	3623
skeletal muscle	3609
vulva	3594
cervix	3436
parathyroid gland	3334
saliva	3306
heart atrial appendage	3152
sigmoid colon	3149
skeletal muscle tissue	3103
aorta	3087
heart left ventricle	3081
esophagus gastroesophageal junction	3080
milk	3066
esophagus muscularis mucosa	3011
skin - not sun exposed (suprapubic)	3008
vagina	2939
skin - sun exposed (lower leg)	2908
tibial nerve	2879
esophagus mucosa	2763
nasopharynx	2657
transverse colon	2621
small intestine - terminal ileum	2592
tibial artery	2592
pituitary gland	2205
coronary artery	2110
minor salivary gland	1750
sclera	1624
blood serum	537
cerebrospinal fluid	240

Supplementary Note 8: IBAQ-based expression profile provided by quantms.org

The distribution of IBAQ-based protein expression in various tissues could be visualised by entering the UniProt accessions of interest in the search field. For example, protein P01721 is reported as only quantified in the spleen tissue from PaxDB and ProteomicsDB. However, we have discovered that protein P01721 is consistently quantified in multiple datasets across various tissues such as the heart, colon, prostate, pancreas, lung, adrenal glandkidney, and stomach, as shown in quantms.org (https://quantms.org/baseline/tissues?protein=P01721). The gene expression resources have expression evidence for the corresponding gene (IGLV6-57 - ENSG00000211640) in more than 93 tissues (e.g. https://www.bgee.org/gene/ENSG00000211640?expression=anat&data_type=RNA_SEQ%2CSC_RNA_SEQ)

Furthermore, multiple proteins with expression profiles could be compared in different tissues by entering multiple UniProt accessions. quantms.org offered an intuitive comparison of protein expression profiles across various tissues.

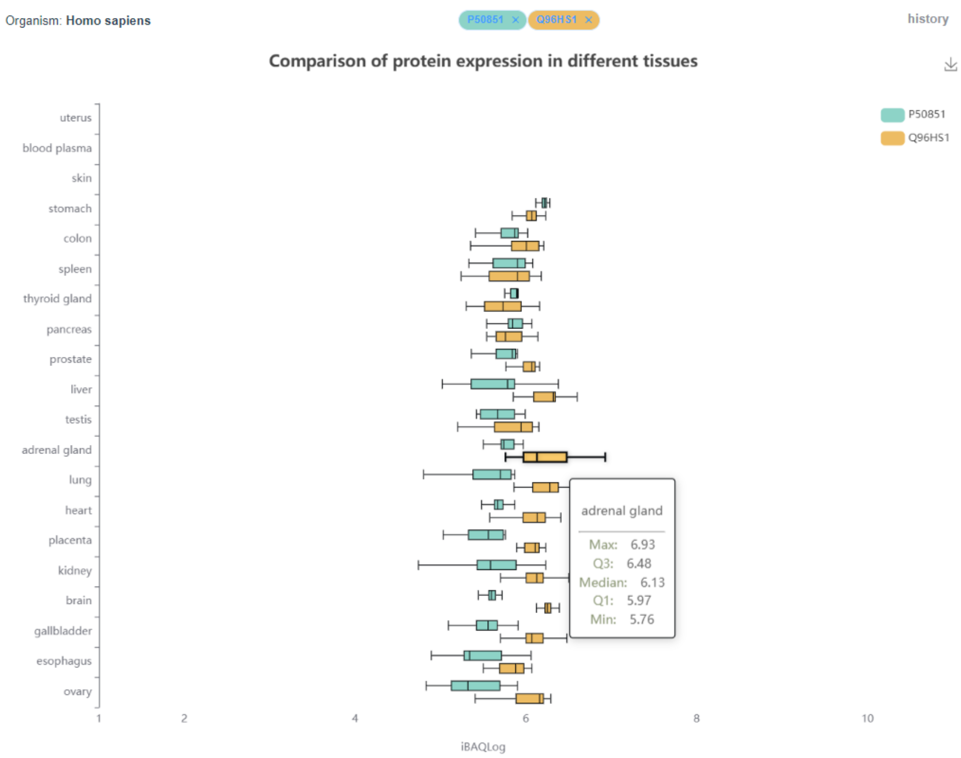

Figure 23: The distribution of protein expression of P50851 and Q96HS1 proteins provided by quantms.org in different sources.

Supplementary Note 9: Quality control using pmultiqc.

Quality control is an essential requirement to create confidence in the generated results [24]. A typical mass spectrometry experiment consists of multiple different phases including sample preparation, liquid chromatography, mass spectrometry, and bioinformatics stages. As part of the quantms workflow, we developed a novel library and web application quality control of the quantms results. pmultiqc (https://github.com/bigbio/pmultiqc) is a Python library for proteomics QC report based on the MultiQC framework [25].

Quantms reports present multiple sections including:

- Experimental Design: Description of samples and the experimental design including, fractions, replicates, and their relationship with the spectra file name.
- HeatMap: A Heat Map containing the distribution of multiple QC metrics including contaminants, peptide intensity, charge, missed cleavages, and identification rate over retention time (RT).

- Summary Table: A summary table resuming the number of peptides, and proteins identified and quantified.
- Number of Peptides Per Protein: A plot with the distribution of peptides identified per protein.
- Spectra Tracking: A table with the number of peptides identified by each search engine.
- Distribution of precursor charges: A plot with the distribution of charge states for unidentified and identified spectra.
- Number of Peaks per MS/MS spectrum: A plot with the distribution of the number of peaks by MS/MS spectra.
- Peak Intensity Distribution: A plot with the distribution of the peak intensity by MS/MS spectra.
- Delta Mass: Delta mass distribution. This plot has been used before by different tools [4, 26, 27] to assess the quality of the identification step.
- Summary of Search Engine Scores: Search engine score distributions, including all scores from the supported and executed search engines (MSGF+ and Comet).

Peptides Quantification Table and Protein Quantification Table: Searching specific peptides and proteins.

pmultiqc provides tables, Peptide and Protein quantitation enabling users to navigate, search and visualize the quantified peptides (**Figure 24**) and proteins (**Figure 25**). Both tables provide general information such as peptide sequence, protein name, best search engine score (for peptides) or the number of peptides per protein. Additionally, the average intensity for each peptide (Figure 24) and protein (**Figure 25**) is calculated using the intensity of the given peptide/protein on each sample. The distribution of intensities by samples is shown for each condition value (e.g., squamous cell carcinoma vs normal).

Searching is possible because quantms data is stored in an SQLite database (https://www.sqlite.org/index.html). The pmultiqc access to the database without needing to be run a server, for the database or the web application. Searching is possible using peptide sequences or protein names and all table columns are sortable.

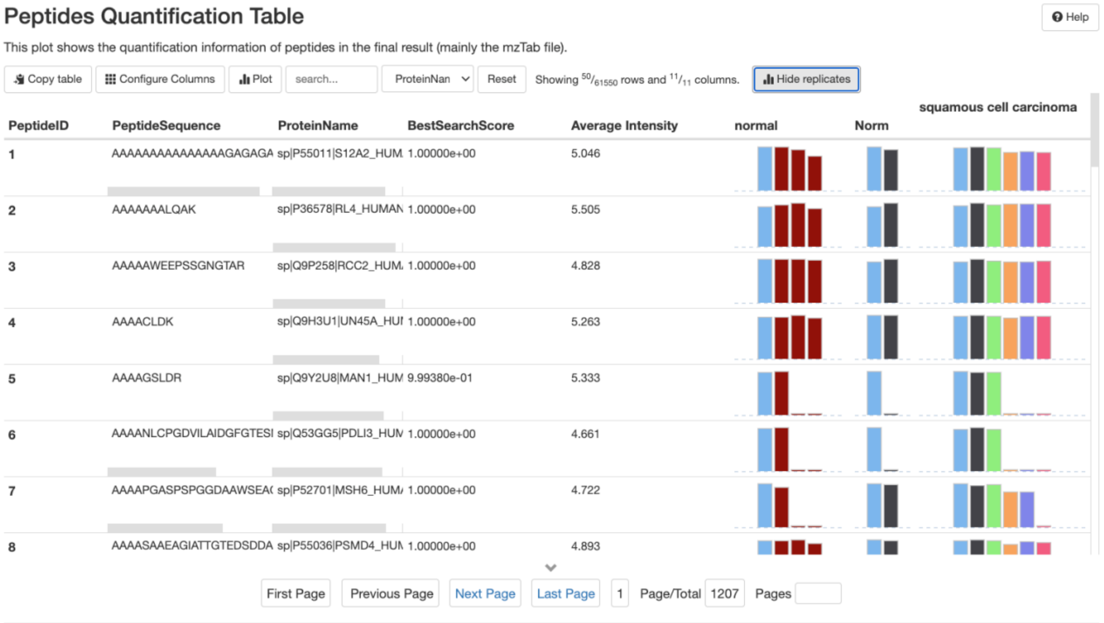

Figure 24: Peptide quantitation table including the following properties: peptide sequence, protein name, best search score, average intensity (across conditions), and intensity for each replicate and condition.

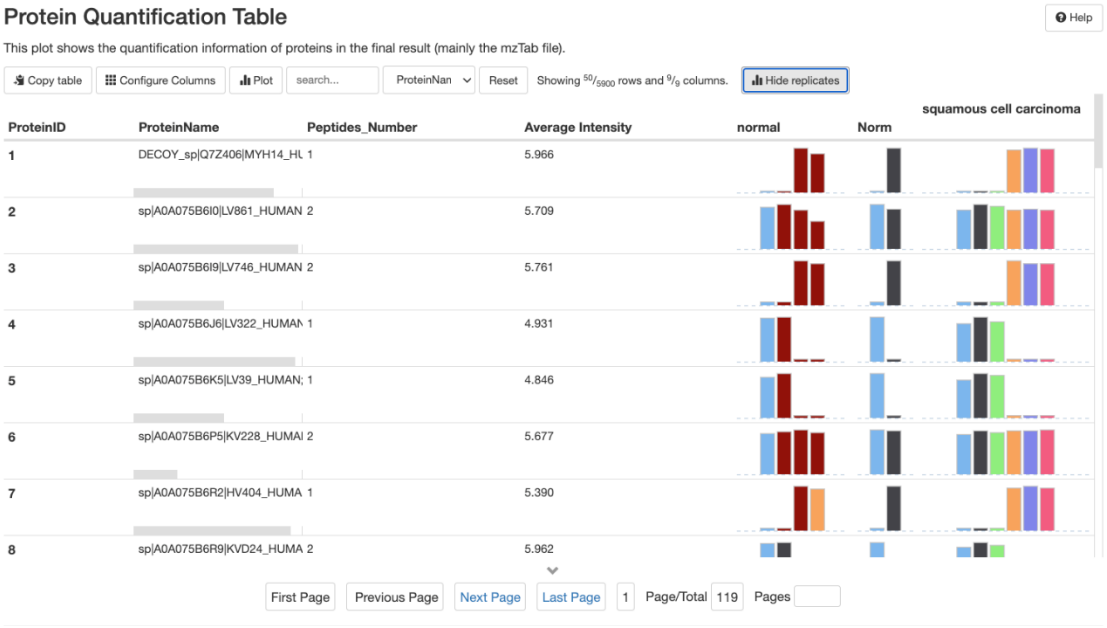

Figure 25: Protein quantitation table including the following properties: protein name, number of peptides, average intensity (across conditions), and intensity for each replicate and condition.

Supplementary Note 10: Implementing workflows on AWS, Google Cloud and other cloud infrastructures.

The quantms is part of an ecosystem of nf-core workflows and pipelines that is based on Nextflow language. Therefore, quantms can be easily run on AWS and other cloud infrastructures. It also provides interfaces like a tower (https://cloud.tower.nf/) that enable running the workflows using web interfaces. One example of how this will look is https://nf-co.re/launch?pipeline=quantms&release=1.2.0 where parameters are translated into a web page interface. We are also part of the nf-tower default community showcase pipelines that you can easily run on free AWS trial credits when logging in for free into nf-tower (https://tower.nf/orgs/community/workspaces/showcase/launchpad/260903829135591). We have made available multiple YouTube training videos in quantms YouTube channel:
https://www.youtube.com/playlist?list=PLMYYkj5pIyVeJZClJloWeo2f97K_gM_Cb

about how to use the workflow in English and Chinese:

- https://www.youtube.com/watch?v=pBzelkgrPgQ
- https://www.youtube.com/watch?v=080me-EEVnU
- https://www.youtube.com/watch?v=w3_bzzfSv7I

We also provided an example to estimate cost powered by nf-tower for the run of one experiment in AWS (https://tower.nf/orgs/community/workspaces/showcase/watch/2UuiO5omjj8lmu). The example is from an LFQ experiment, consisting of six raw files. The total running time was 18 minutes, with 0.4 CPU hours used and a total memory consumption of 6.87 GB. The cost estimation was 0.012 dollars. AWS is billed by time, but different clouds may have different billing methods.

References

1. Kim, S. & Pevzner, P.A. MS-GF+ makes progress towards a universal database search tool for proteomics. *Nat Commun* **5**, 5277 (2014).
2. Eng, J.K., Jahan, T.A. & Hoopmann, M.R. Comet: an open-source MS/MS sequence database search tool. *Proteomics* **13**, 22-24 (2013).
3. Audain, E. et al. In-depth analysis of protein inference algorithms using multiple search engines and well-defined metrics. *J Proteomics* **150**, 170-182 (2017).
4. Vaudel, M. et al. PeptideShaker enables reanalysis of MS-derived proteomics data sets. *Nat Biotechnol* **33**, 22-24 (2015).
5. Umer, H.M. et al. Generation of ENSEMBL-based proteogenomics databases boosts the identification of non-canonical peptides. *Bioinformatics* (2021).
6. Choi, M. et al. MassIVE.quant: a community resource of quantitative mass spectrometry-based proteomics datasets. *Nat Methods* **17**, 981-984 (2020).
7. Lautenbacher, L. et al. ProteomicsDB: toward a FAIR open-source resource for life-science research. *Nucleic Acids Res* **50**, D1541-D1552 (2022).
8. Perez-Riverol, Y. et al. The PRIDE database resources in 2022: a hub for mass spectrometry-based proteomics evidences. *Nucleic Acids Res* **50**, D543-D552 (2022).

9. Prakash, A. et al. Integrated View of Baseline Protein Expression in Human Tissues. *J Proteome Res* (2022).
10. Bai, M., Deng, J., Dai, C., Pfeuffer, J. & Perez-Riverol, Y. LFQ-based peptide and protein intensity downstream analysis. (2022).
11. Ramus, C. et al. Benchmarking quantitative label-free LC-MS data processing workflows using a complex spiked proteomic standard dataset. *J Proteomics* **132**, 51-62 (2016).
12. Ramus, C. et al. Spiked proteomic standard dataset for testing label-free quantitative software and statistical methods. *Data Brief* **6**, 286-294 (2016).
13. Palomba, A. et al. Comparative Evaluation of MaxQuant and Proteome Discoverer MS1-Based Protein Quantification Tools. *J Proteome Res* **20**, 3497-3507 (2021).
14. Valikangas, T., Suomi, T. & Elo, L.L. A comprehensive evaluation of popular proteomics software workflows for label-free proteome quantification and imputation. *Brief Bioinform* **19**, 1344-1355 (2018).
15. Pino, L.K. et al. The Skyline ecosystem: Informatics for quantitative mass spectrometry proteomics. *Mass Spectrom Rev* **39**, 229-244 (2020).
16. Bouyssie, D. et al. Mascot file parsing and quantification (MFPaQ), a new software to parse, validate, and quantify proteomics data generated by ICAT and SILAC mass spectrometric analyses: application to the proteomics study of membrane proteins from primary human endothelial cells. *Mol Cell Proteomics* **6**, 1621-1637 (2007).
17. D'Angelo, G. et al. Statistical Models for the Analysis of Isobaric Tags Multiplexed Quantitative Proteomics. *J Proteome Res* **16**, 3124-3136 (2017).
18. Paulo, J.A., O'Connell, J.D., Gaun, A. & Gygi, S.P. Proteome-wide quantitative multiplexed profiling of protein expression: carbon-source dependency in Saccharomyces cerevisiae. *Mol Biol Cell* **26**, 4063-4074 (2015).
19. D'Aguanno, S. et al. p63 isoforms regulate metabolism of cancer stem cells. *J Proteome Res* **13**, 2120-2136 (2014).
20. Huang, T. et al. MSstatsTMT: Statistical Detection of Differentially Abundant Proteins in Experiments with Isobaric Labeling and Multiple Mixtures. *Mol Cell Proteomics* **19**, 1706-1723 (2020).
21. Griss, J., Vinterhalter, G. & Schwammle, V. IsoProt: A Complete and Reproducible Workflow To Analyze iTRAQ/TMT Experiments. *J Proteome Res* **18**, 1751-1759 (2019).
22. Gotti, C. et al. Extensive and Accurate Benchmarking of DIA Acquisition Methods and Software Tools Using a Complex Proteomic Standard. *J Proteome Res* **20**, 4801-4814 (2021).
23. Dai, C. et al. A proteomics sample metadata representation for multiomics integration and big data analysis. *Nat Commun* **12**, 5854 (2021).
24. Bittremieux, W. et al. Quality control in mass spectrometry-based proteomics. *Mass Spectrom Rev* **37**, 697-711 (2018).

25. Ewels, P., Magnusson, M., Lundin, S. & Kaller, M. MultiQC: summarize analysis results for multiple tools and samples in a single report. *Bioinformatics* **32**, 3047-3048 (2016).
26. Perez-Riverol, Y. et al. PRIDE Inspector Toolsuite: Moving Toward a Universal Visualization Tool for Proteomics Data Standard Formats and Quality Assessment of ProteomeXchange Datasets. *Mol Cell Proteomics* **15**, 305-317 (2016).
27. Wang, R. et al. PRIDE Inspector: a tool to visualize and validate MS proteomics data. *Nat Biotechnol* **30**, 135-137 (2012).
